# Upregulation of RNA cap methyltransferase RNMT drives ribosome biogenesis during T cell activation

**DOI:** 10.1093/nar/gkab465

**Published:** 2021-06-14

**Authors:** Alison Galloway, Aneesa Kaskar, Dimitrinka Ditsova, Abdelmadjid Atrih, Harunori Yoshikawa, Carolina Gomez-Moreira, Olga Suska, Marcin Warminski, Renata Grzela, Angus I Lamond, Edward Darzynkiewicz, Jacek Jemielity, Victoria H Cowling

**Affiliations:** Centre for Gene Regulation and Expression, School of Life Sciences, University of Dundee, Dow Street, Dundee DD1 5EH, UK; Centre for Gene Regulation and Expression, School of Life Sciences, University of Dundee, Dow Street, Dundee DD1 5EH, UK; Centre for Gene Regulation and Expression, School of Life Sciences, University of Dundee, Dow Street, Dundee DD1 5EH, UK; FingerPrints Proteomics Facility, School of Life Sciences, University of Dundee, Dundee, DD1 5EH, UK; Centre for Gene Regulation and Expression, School of Life Sciences, University of Dundee, Dow Street, Dundee DD1 5EH, UK; Centre for Gene Regulation and Expression, School of Life Sciences, University of Dundee, Dow Street, Dundee DD1 5EH, UK; Centre for Gene Regulation and Expression, School of Life Sciences, University of Dundee, Dow Street, Dundee DD1 5EH, UK; Centre of New Technologies, University of Warsaw, 02-097 Warsaw, Poland; Centre of New Technologies, University of Warsaw, 02-097 Warsaw, and Division of Physics, 02-093 Warsaw, Poland; Centre for Gene Regulation and Expression, School of Life Sciences, University of Dundee, Dow Street, Dundee DD1 5EH, UK; Centre of New Technologies, University of Warsaw, 02-097 Warsaw, and Division of Physics, 02-093 Warsaw, Poland; Centre of New Technologies, University of Warsaw, 02-097 Warsaw, Poland; Centre for Gene Regulation and Expression, School of Life Sciences, University of Dundee, Dow Street, Dundee DD1 5EH, UK

## Abstract

The ^m7^G cap is ubiquitous on RNAPII-transcribed RNA and has fundamental roles in eukaryotic gene expression, however its *in vivo* role in mammals has remained unknown. Here, we identified the ^m7^G cap methyltransferase, RNMT, as a key mediator of T cell activation, which specifically regulates ribosome production. During T cell activation, induction of mRNA expression and ribosome biogenesis drives metabolic reprogramming, rapid proliferation and differentiation generating effector populations. We report that RNMT is induced by T cell receptor (TCR) stimulation and co-ordinates the mRNA, snoRNA and rRNA production required for ribosome biogenesis. Using transcriptomic and proteomic analyses, we demonstrate that RNMT selectively regulates the expression of terminal polypyrimidine tract (TOP) mRNAs, targets of the ^m7^G-cap binding protein LARP1. The expression of LARP1 targets and snoRNAs involved in ribosome biogenesis is selectively compromised in *Rnmt* cKO CD4 T cells resulting in decreased ribosome synthesis, reduced translation rates and proliferation failure. By enhancing ribosome abundance, upregulation of RNMT co-ordinates mRNA capping and processing with increased translational capacity during T cell activation.

## INTRODUCTION

T cells have major roles in adaptive immunity; cytotoxic CD8 T cells directly kill infected or cancerous cells by the transfer of proteolytic enzymes and CD4 helper T cells co-ordinate the actions of other immune cells by the secretion of cytokines and factors. Following development in the thymus, naïve T cells circulate through the blood and reside in the lymphoid organs, quiescent but poised to respond to the recognition of an antigen through their cognate T cell receptor (TCR). Strong TCR signalling drives T cell activation, inducing cell growth, proliferation and differentiation into effector T cells. This requires the reprogramming of cellular metabolism (1,2) and reshaping of the proteome (3,4), which are co-ordinated by global and gene-specific increases in transcription and translation (5–7). Critical to activation, T cell receptor (TCR) stimulation induces ribosome biogenesis, producing the protein synthesis capacity required for rapid proliferation and effector molecule synthesis (8–10).

**Figure 1.**
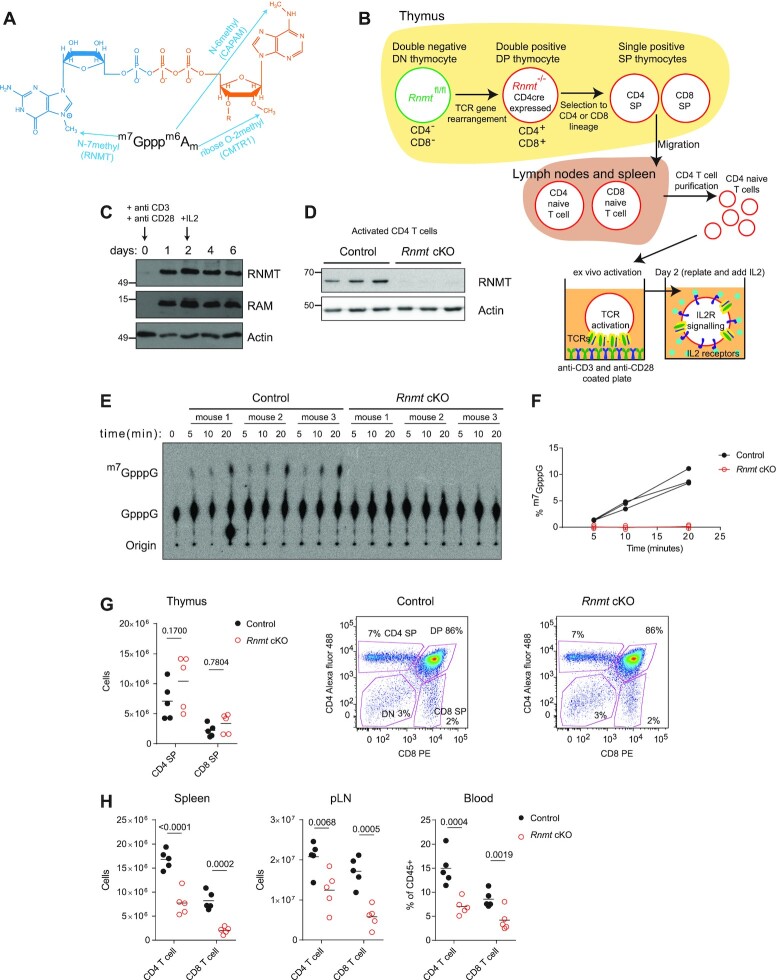
RNMT is upregulated following T cell activation (**A**) ^m7^Gppp^m6^A_m_ cap structure with methyltransferases. (**B**) The *Rnmt* cKO T cell model: mature and progenitor T cell populations are shown with the stage of *Rnmt* deletion and *ex-vivo* activation protocol. (**C**) Control CD4 T cells were activated, after 2 days IL2 was added. Western blot analysis of RNMT and RAM expression. (**D**) RNMT expression in 20 h-activated *Rnmt* cKO (*n* = 3) and control (*n* = 3) CD4 T cells. Equivalent cell numbers analysed. (E, F) Cap methyltransferase activity in 1.6 × 10^5^ control (*n* = 3) and *Rnmt* cKO (*n* = 3) activated CD4 T cells. GpppG capped RNA was incubated with the T cell extracts for the indicated times and the proportion of caps converted to ^m7^GpppG was determined. (**E**) radiograph. (**F**) quantification. (**G**) FACS analysis of control (*n* = 5) and *Rnmt* cKO (*n* = 5) thymi. Quantification of single positive (SP) cells and representative plots displaying live cells. Population names on control plot. Dots indicate biological replicates, lines indicate means, *P* values from ANOVA with Sidak's multiple comparisons test throughout. (**H**) FACS quantification of CD4 and CD8 T cells in spleens, peripheral lymph nodes (pLN), and percentage in blood from control (*n* = 5) and *Rnmt* cKO (*n* = 5) mice. Figures are representative of (C, D) three and (G, H) two experiments.

Following transcription, RNA polymerase II (RNAPII)-transcribed RNAs, including mRNA, miRNA, lncRNA, snRNA and snoRNA precursors, are modified by the addition of a 7-methylguanosine (^m7^G) RNA cap (11,12) (Figure 1A). ^m7^G cap formation involves cap guanosine addition to the 5′ end of nascent RNA, catalysed by the capping enzyme RNGTT (RNA guanylyltransferase and 5′-phosphatase), followed by cap guanosine N-7 methylation catalysed by RNMT (RNA guanine-7 methyltransferase) (12). RNMT has an activating subunit, RAM (RNMT-activating miniprotein/RAMAC), which increases methyltransferase activity and recruits RNA to the complex (13,14). Most non-coding RNAs lose their ^m7^G cap during maturation by cleavage or further modification to ^m2,2,7^G trimethylguanosine.

The ^m7^G cap has fundamental functions; it recruits RNA processing, modification and translation factors, protects RNA from degradation and identifies it as self by blocking the binding of viral RNA sensing receptors (12,15). The discovery of regulation of the capping enzymes and the identification of variants of the cap binding complexes reveals the RNA cap as a key integration point of signalling pathways and gene expression (12,16–20). Such co- and post-transcriptional regulation mechanisms are increasingly recognised as integral to immune responses (21).

Here, we report that the ^m7^G cap methyltransferase, RNMT, is induced during T cell activation and is required for the substantial increases in RNA production, processing and translation which support T cell expansion. Unexpectedly, RNMT has a gene-specific impact, controlling expression of a subset of RNAs including the terminal polypyrimidine tract (TOP) mRNAs encoding ribosomal proteins, ribosome biogenesis factors and translation factors as well as several non-coding snoRNAs involved in ribosome biogenesis. TOP RNAs are stabilised by a specialised cap binding protein, La-related protein 1 (LARP1), which has a preference for binding to ^m7^G-capped TOP RNAs (22–25). We identified the LARP1 binding sites in naïve CD4 T cells and found a correlation between cap-adjacent LARP1 binding and reduced RNA expression in *Rnmt* cKO CD4 T cells. Expression of the proteins encoded by LARP1-bound RNAs were reduced in activated *Rnmt* cKO CD4 T cells, particularly ribosomal proteins and proteins mediating ribosome biogenesis and translation. Thus, we demonstrate that the RNMT RNA regulon co-ordinates the mRNA synthesis and ribosome biogenesis critical for T cell activation.

## MATERIALS AND METHODS

### Mice

*Rnmt* fl/fl mice with loxP sites flanking exon 3 of *Rnmt* were sourced from Taconic Artemis Gmbh. CD4-Cre (Tg(Cd4-cre)1Cwi) (26) mice were gifted by Doreen Cantrell. Mice were maintained on a C57B6/J background in the Biological Resource Unit at the University of Dundee using procedures approved by the University Ethical Review Committee and under the authorization of the UK Home Office Animals (Scientific Procedures) Act 1986.

### Preparation of cells

Lymph nodes (inguinal, brachial, axillary, superficial cervical, mesenteric, lumbar, caudal), spleens and thymi were dissected from mice, and mashed through a 70 μm cell strainer (Falcon) to prepare cell suspensions. Blood was taken by cardiac puncture (following confirmation of death) and diluted in RPMI medium, red blood cells were lysed with ACK buffer (150 mM NH_4_Cl, 10 mM KHCO_3_, 0.1 mM Na_2_EDTA). Cells were counted either using the BD FACSVerse (BD Biosciences) or Novocyte (Acea Biosciences) flow cytometer, by haemocytometer (Fastread counting slides from Immune systems) or using AccuCheck counting beads (Thermo Fisher Scientific) in combination with a BD LSRFortessa (BD Biosciences) flow cytometer.

### Western blotting

For analysis of RPs, LARP1 and RAM in supplemental figure 1 M T cells were lysed directly in Laemmli buffer (50 mM Tris pH 6.8, 2% SDS, 10% glycerol, 100 mM DTT, bromophenol blue). For analysis of RNAPII, RNMT and RAM, T cells were lysed in F-buffer (10 mM Tris–HCl pH 7.05, 50 mM NaCl, 0.5% Triton X-10). For RNAPII analysis, lysates were sonicated 3 × 30 s on a Bioruptor (Diagenode) at medium intensity. Equivalent cell numbers were resolved by SDS-PAGE. For standard western blots, proteins were transferred onto PVDF membranes (Millipore) with Tris-glycine buffer (25 mM Tris, 190 mM glycine, 20% methanol). For analysis of ribosomal proteins the higher pH CAPs buffer (10 mM CAPs 20% methanol) was used. Membranes were incubated with primary antibodies (Supplementary Table S1), followed by the relevant HRP-conjugated secondary antibodies (Supplementary Table S1) and developed with Pierce Super signal ECL (Thermo Fisher scientific), visualised with X-ray film or using an ImageQuant LAS 4000 (GE healthcare). Western blots were quantified using NIH ImageJ software.

### ^m7^GTP binding assay

Jurkat cells were cultured in RPMI media, supplemented with non-essential amino acids (Gibco) and sodium pyruvate (Gibco). Jurkat cell pellets were lysed using Buffer B (10 mM HEPES pH 7.4, 1 mM MgCl_2_, 10 mM NaCl, 50 mM NaF, 0.5% NP-40). Lysates were pre-cleared with Protein A-Sepharose beads (Generon, PCA-125) for 30 min at 4°C, rotating. Lysate were split into equal parts, each incubated with 40 ul ^m7^GTP-Agarose (Jena Bioscience) or GTP-Sepharose for 2 h at 4°C. Beads were washed with Buffer B. After washes, beads were re-suspended in LDS sample buffer (Novex) with 0.1 M DTT diluted in Buffer B. ^m7^GTP and GTP pulldown samples were resolved on the SDS-PAGE gels with SDS Running Buffer (25 mM Tris, 250 mM glycine, 0.1% SDS), then transferred to PVDF membranes, which were probed with rabbit anti-LARP1 antibody (ProteinTech).

### Flow cytometry

Cells were incubated with labelled antibodies (Supplementary Table S1) and FC block (anti CD16/32, Biolegend) in FACS buffer (PBS + 2% FCS (Gibco)). Dead cells were labelled with 0.1 μg/ml DAPI or Fixable Viability Dye eFluor 780 (eBioscience). For apoptosis, cells were stained with annexin V APC (Biolegend) in annexin V buffer (10 mM HEPES pH 7.4, 140 mM NaCl, 2.5 mM CaCl_2_, 4 mM KCl, 0.75 mM MgCl_2_). For translating ribosomes, cells were incubated with 1 ug/ml puromycin for 10 min at 37°C in T cell culture medium, stained with Fixable Viability Dye eFluor 780, fixed with 1% PFA in PBS (Santa Cruz Biotech) on ice, and permeablised and stained for puromycin and surface markers in saponin buffer (PBS+ 5% FCS + 0.1% saponin) (27). For cell cycle analysis, cells were fixed in 4% PFA, permeablised in ice cold 90% methanol 10% PBS, and stained with antibodies then 1 μg/ml DAPI in FACS buffer. Data was collected using either a BD FACSVerse, or LSR Fortessa and analyzed using FlowJo10 (BD).

### T cell culture

To measure proliferation, apoptosis, puromycin incorporation and forward scatter, lymph node cells were cultured in T cell culture medium (RPMI + 10% heat inactivated FCS + pen/strep + 50 μM 2ME) with 0.5 ug/ml Ultra-LEAF purified anti-mouse CD3ϵ antibody (clone 145-2C11, Biolegend), and 0.5 ug/ml Ultra-LEAF purified anti-mouse CD28 antibody (clone 37.51, Biolegend). 20ng/ml IL-2 (proleukin, Norvatis) was added on day 2. For analysis of protein or RNA in CD4 T cells, magnet sorted CD4 T cells were cultured in T cell culture medium on anti-CD3 (5 μg/ml)/anti-CD28 (1 μg/ml) coated plates.

### Preparation of CD4 T cells

To purify T cells, for most experiments CD4 T cells were magnet sorted using EasySep mouse CD4 T cell isolation kit (Stemcell Technologies), for the ribosome footprinting experiment the mouse naïve CD4 T cell kit was used, for CAP-MAP, eCLIP, and Ψ-seq the Miltenyi mouse CD4 T cell kit was used. For proteomics and naïve T cell RNA sequencing analysis, naïve CD4 T cells were FACS sorted using the BD Influx (BD Biosciences), purities were > 98%.

### RNA assays

N-7 cap guanosine methylation assay was performed according to (14). The *in vivo* cap methylation assay was performed according to (28). More details in supplemental materials.

RNA was extracted using Tri-reagent (Sigma) or Trizol (Thermo Fisher scientific) and quantified by Nanodrop (Thermo Fisher scientific). For qPCR, cDNA synthesised using iScript (BioRad), then qPCRs performed on a BioRad CFX384 using ssoFast Eva Green supermix (Bio-rad) and primers in Supplementary Table S1. For mRNA extraction a Dynabeads mRNA DIRECT™ Purification kit was used on total RNA with two rounds of purification. mRNA was quantified using the Qubit RNA HS Assay Kit (Thermo Fisher scientific). For ^3^H uridine labelling of RNA, cells were cultured with 0.37 MBq [5,6–^3^H] uridine/ml medium, RNA and mRNA extracted and scintillation counts measured using Optiphase Hisafe3 scintillation fluid (Perkin Elmer) and a Tri-Carb 4910TR scintillation counter (Perkin Elmer).

For northern blotting, 1 μg total RNA was analysed according to the previous report (29) with methylene blue staining of 28S and 18S rRNAs for loading control. Oligonucleotides probes for pre-rRNA detection were: 5′-ITS1, 5′-GCTCCTCCACAGTCTCCCGTTTAATGATC-3′; ITS1-1b, 5′-CTCTCACCTCACTCCAGACACCTCGCTCCA-3′; ITS2-1, 5′-ACCCACCGCAGCGGGTGACGCGATTGATCG−3′ (30,31). 3′-end biotin-labelled oligonucleotides were synthesized by Eurofins Genomics.

### DNA qPCR

DNA was isolated from CD4 T cells using a DNA blood and tissue kit (Qiagen). qPCR was performed as described for cDNA.

### RNA sequencing

Naïve T cell transcriptome libraries were prepared by the Tayside Centre for Genomic Analysis using an Illumina Truseq kit with Ribozero Gold rRNA depletion. 20 h activated T cell cytoplasmic transcriptome and ribosome footprint libraries were prepared using the Illumina Truseq Ribo Profile (mammalian) kit. eCLIP was carried out as described in the seCLIP protocol with a few modifications (32). Pseudouridine-seq was carried out according to the Ψ-seq protocol (33). CD4 T cell RNAPII CHIPseq (RNAPII: GSM1903984, input: GSM1903982) (34), and RAM knockdown HeLa cell RNAseq (GSE87767) (16), datasets were obtained from NCBI Geo. Library preparation methods and analysis are further detailed in supplemental materials and methods.

### Mass spectrometry

CAP-MAP mass spec analysis was carried out as described previously. TMT proteomics was performed as described previously (35). Ribo Mega-SEC was performed as described previously (29). Further methods and analyses are detailed in supplemental materials and methods.

### Statistical analysis

Excluding ‘omics’ data, statistical analyses and charts were generated in Graphpad Prism. Where used, t-tests were double sided and unpaired.

### Further information

Further detailed protocol information is available in the supplemental materials. Antibodies, primers and other key reagents are listed in Supplementary Table S1.

## RESULTS

### RNMT is upregulated following T cell activation

Following T cell activation, major increases in transcription and translation are likely to require increased production of the ^m7^G cap to protect, process and translate the nascent mRNA. Consistent with this, we found that the ^m7^G cap methyltransferase, RNMT, and co-factor, RAM, were upregulated downstream of T cell receptor (TCR) signalling, induced using crosslinking antibody stimulation, and maintained throughout CD4 and CD8 T cell activation in the presence of the stimulatory cytokine interleukin 2 (IL2) (Figure 1B, C, Supplementary Figure S1A–E). To assess the role of RNMT in T cells, we crossed mice with *Rnmt* floxed alleles to mice expressing CD4-Cre to delete *Rnmt* at the double positive (DP) stage of T cell development (Figure 1B, Supplementary Figure S1F). These cells are the developmental precursors to the mature T cells that are the subject of this study (26). In *Rnmt*^fl/fl^ CD4-Cre mice (*Rnmt* cKO, conditional knock-out), RNMT protein was gradually lost during thymic T cell development and was minimal in naïve or activated lymph node CD4 T cells compared to the equivalent cells in *Rnmt*^fl/fl^ mice (controls) (Figure 1D, Supplementary Figure S1G–J). Deletion of *Rnmt* exon 3 in genomic DNA and loss of RNA reads aligning to *Rnmt* exon 3 was confirmed in *Rnmt* cKO naïve CD4 T cells (Supplementary Figure S1K, L). The expression of RNMT and RAM are co-dependent in other cell lineages, and RAM was repressed in activated *Rnmt* cKO CD4 T cells (Supplementary Figure S1M-N) (36). ^m7^G cap methyltransferase activity was undetectable in *Rnmt* cKO in activated (Figure 1E, F) or naïve (Supplementary Figure S1O, P) CD4 T cell extracts, confirming the loss of RNMT protein and the absence of any entirely redundant ^m7^G cap methyltransferase. *Rnmt* cKOs had normal numbers of DP, CD4 single positive (SP) and CD8 SP in the thymus compared to controls, but ∼50% less CD4 and CD8 T cells in the peripheral lymph nodes, spleen and blood (Figure 1G-H). The proportions of central or effector memory CD4 and CD8 T cells were equivalent in *Rnmt* cKO mice and controls (Supplementary Figure S1Q). Thus, loss of *Rnmt* has a moderate impact on T cell homeostasis.

### Cap guanosine N-7 methylation is reduced in *Rnmt* cKO T cells

**Figure 2.**
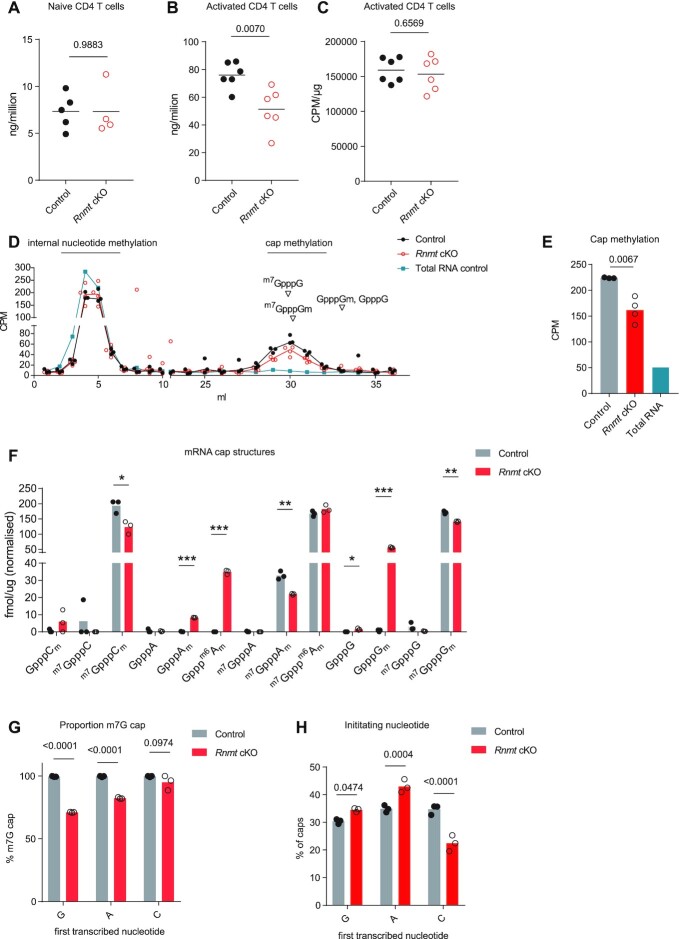
*Rnmt* cKO T cells have reduced mRNA cap methylation and C-initiating mRNA (**A**) dT-purified mRNA/cell from control (*n* = 5) and *Rnmt* cKO (*n* = 4) naïve CD4 T cells. Dots represent biological replicates, bars indicate means. *P* values from Student's *t*-test. (B, C) 20 h-activated CD4 T cells were incubated with ^3^H uridine for 4 h. mRNA was dT-purified from control (*n* = 6) and *Rnmt* cKO (*n* = 6) cells. (**B**) mRNA per million cells. (**C**) CPM per μg mRNA. Dots represent biological replicates, bars indicate means. P values from Student's *t*-test. Data combined from two experiments. (D, E) CD4 T cells activated for 20 h were ^3^H-labelled on methyl groups for 2.25 h. Nucleotides from total RNA from control cells (*n* = 1), and mRNA from control (*n* = 3) and *Rnmt* cKO cells (*n* = 4) were resolved by chromatography. Equivalent RNA loaded. (**D**) Counts per minute (CPM) presented. Elution volumes of cap dinucleotides and nucleotides determined using standards. Points represent replicates, lines join medians. (**E**) CPM from cap dinucleotides, fractions 28–34 ml. Dots represent biological replicates, bars indicate mean. *P* value from Student's *t*-test. (F, H) CAP MAP analysis in *Rnmt* cKO and control 20 hour activated CD4 T cells. (**F**) Quantitation of cap structures. *P* values from multiple t-tests corrected with FDR approach * *P* <0.050, ** *P* < 0.010, ***P < 0.001. (**G**) Proportion of caps with ^m7^G structures. *P* values from ANOVA with Sidak's multiple comparisons test. (**H**) Proportion of caps initiating with each nucleotide. *P* values from ANOVA with Sidak's multiple comparisons test. Dots represent biological replicates, bars indicate mean.

Although ^m7^G cap methyltransferase activity was minimal in *Rnmt* cKO CD4 T cell extracts (Figure 1E-F, Supplementary Figure S1O, P), it was important to determine the extent of mRNA and cap synthesis *in vivo*. The mRNA content per cell was unchanged in *Rnmt* cKO naïve CD4 T cells, but the induction of mRNA synthesis following activation was impaired suggesting that RNMT and cap formation is more critical for RNA production in activated T cells (Figure 2A, B). Nevertheless, uridine incorporation into mRNA was equivalent on a per-mRNA basis at 20 h following activation, indicating comparable net mRNA synthesis/degradation at this timepoint (Figure 2C). We determined the rate of endogenous mRNA cap methylation in activated CD4 T cells by culturing them with ^3^H-methionine, which is converted to the methyl donor, *S*-adenosyl methionine (SAM), leading to ^3^H-labelling of methyltransferase substrates (28). Methylation of internal nucleotides did not change in *Rnmt* cKO CD4 T cells compared to controls, consistent with no change in net mRNA synthesis/degradation (Figure 2D, Supplementary Figure S2A). Cap methylation, which includes both ^m7^G and first nucleotide methylation, was reduced by ∼30% in *Rnmt* cKO CD4 T cells (Figure 2D, E). Given that we do not expect first nucleotide methylation to be affected, this indicates a significant reduction in the rate of ^m7^G formation.

The cap structures present on mRNA from activated T cells were quantified by CAP-MAP mass spectrometry (37). *Rnmt* cKO CD4 T cells had significantly more cap structures which lack ^m7^G: GpppG_m_, Gppp^m6^A_m_, and GpppA_m_, and significantly less mature ^m7^G-cap structures: ^m7^GpppC_m_, ^m7^GpppG_m_ and ^m7^GpppA_m_ than controls (Figure 2F). Our protocols do not detect ^m7^GpppU_m_. Over 99% of control CD4 T cell mRNA had a ^m7^G cap structure, whereas in *Rnmt* cKOs ^m7^G-caps were reduced by 28% on mRNAs initiating with guanosine and by 17% on mRNAs initiating with adenosine (Figure 2G). Thus in *Rnmt* cKO CD4 T cells, ^m7^G-caps are synthesised at a lower rate, their steady state concentration is lower, but the modification is still abundant, indicating selection for mRNA bearing a complete ^m7^G cap structure. Interestingly, *Rnmt* cKOs had a 35% decrease in mRNA initiating with cytosine, but ∼95% of this mRNA had an ^m7^G cap (Figure 2G, H). This implies that the incomplete cap generated when the first transcribed nucleotide is cytosine (GpppCm), is the least tolerated incomplete cap structure, leading to loss of RNA initiating with a C on *Rnmt* deletion.

### *Rnmt* deletion has a gene-specific impact including selective loss of TOP mRNAs

**Figure 3.**
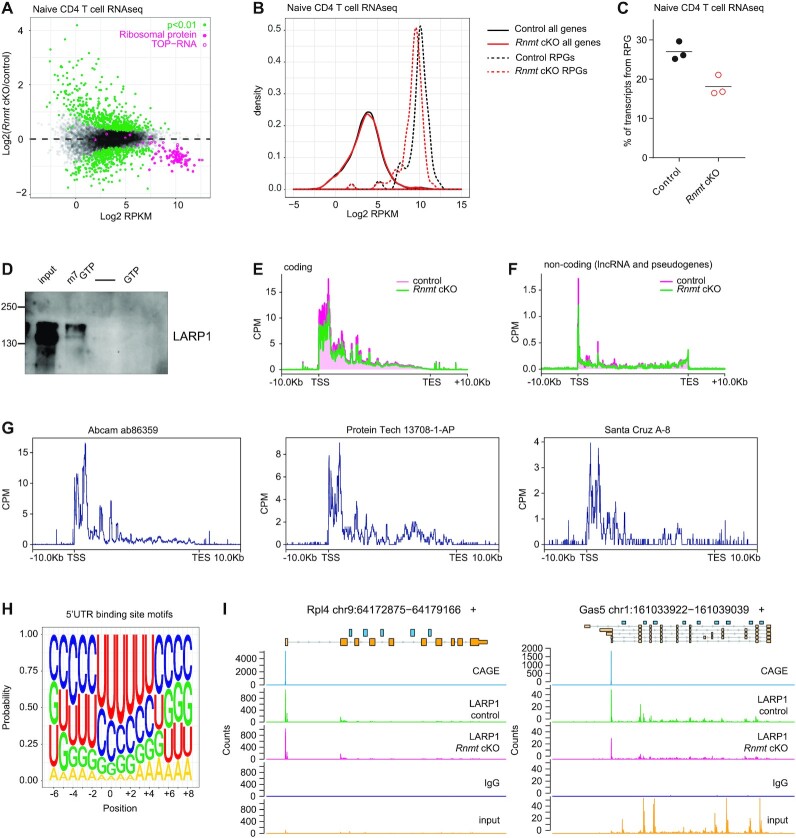
LARP1 target RP mRNAs are sensitive to loss of *Rnmt* (A–C) RNAseq analysis of control (*n* = 3) and *Rnmt* cKO (*n* = 3) naïve CD4 T cells. (**A**) MA plot of RNA expression. Dots represent genes. Reads per million mapped reads (RPKM) on x-axis. Control and *Rnmt* cKO samples were compared using EdgeR exact test and adjusted *P*-value used. Ribosomal protein genes and other TOP-RNA genes indicated. (**B**) Distribution of RPKM within all genes or ribosome protein genes (RPGs) in control and *Rnmt* cKO naïve CD4 T cell RNAseq data. (**C**) % of transcripts from RPG calculated using RPKM as an approximation of transcript abundance. (**D**) Western blot analysis of LARP1 from Jurkat cell lysates enriched on ^m7^GTP- or GTP-agarose beads. Data representative of three experiments. (**E-I**) LARP1 eCLIP of control and *Rnmt* cKO naïve CD4 T cells. Distribution of reads as counts per million (CPM) across protein-coding transcripts (**E**) and non-coding genes (pseudogene and lncRNA) (**F**) with LARP1 binding sites. (**G**) Distribution of reads from LARP1 eCLIP on control naïve CD4 T cells as counts per million (CPM) along target transcripts for three different anti-LARP1 antibodies. (All other data is Abcam ab86359 antibody which was selected for further analysis). (**H**) Nucleotide composition surrounding LARP1 binding sites in 5′UTRs. (**I**) Reads from anti-LARP1 antibody purified transcripts, isotype control antibody, and size matched input reads aligning to two example LARP1 target transcripts. Genes annotated, dark yellow = protein coding transcripts, light yellow = non-coding genes, blue = snoRNA. CD4 T cell CAGE data from FANTOM 5 project shown for reference. Counts are the number of reads starting at that position; read starts are expected to be 1nt downstream of the crosslink site.

To determine the impact of *Rnmt* deletion on RNA expression we performed RNA sequencing (seq) analysis on naïve CD4 T cells. Of 11 219 genes which passed analysis thresholds, 753 were significantly reduced and 845 were significantly increased in *Rnmt* cKOs compared to controls (Figure 3A, Supplementary Table S2). Pathway analysis indicated that the downregulated transcripts were enriched for ribosomal protein genes, with 58 of the 69 genes in this pathway significantly decreased in *Rnmt* cKOs (Figure 3A, Supplementary Figure S2B). Ribosomal protein (RP) genes were the only significantly enriched pathway among the RNMT-sensitive genes, so we focussed further analysis on this group. Differential expression of selected genes was confirmed by RT-qPCR (Supplementary Figure S2C). RP transcripts were highly abundant in naïve CD4 T cells: using the reads per kilobase per million (RPKM) as an approximation of transcript abundance they constituted ∼27% of the protein coding transcriptome, which reduced to ∼18% in *Rnmt* cKOs (Figure 3B, C). In HeLa cells, genes with highest RNAPII occupancy have the highest sensitivity to knockdown (KD) of RNMT-RAM (16). Analysis of published RNAPII ChIP data indicated that RP genes also have high RNAPII occupancy in naïve CD4 T cells (34), however, other transcripts with high RNAPII occupancy remain unaffected in *Rnmt* cKO CD4 T cells (Supplementary Figure S2D), indicating that high RNAPII is not predictive of RNMT dependence in CD4 T cells. Furthermore, unlike in HeLa cells, RNAPII CTD (C-terminal domain) Ser-5 and Ser-2 phosphorylation and net mRNA synthesis/degradation were not RNMT-dependent suggesting that transcription is not significantly regulated through RNMT in CD4 T cells (Figure 2C, Supplementary Figure S2E, F).

Transcripts encoding RPs and certain translation factors are members of a family of mRNAs initiating with cytosine followed by a terminal oligopyrimide (TOP) motif in their 5′ untranslated region (UTR). TOP RNAs bind to a specialised cap binding protein, La-related protein 1 (LARP1), which enhances their stability and represses their translation in MTOR and CDK1-regulated mechanisms that coordinate ribosome biogenesis with cell growth and circadian rhythms (24,38–45). Canonical cap binding proteins such as EIF4E of the EIF4F complex and NCBP2 of the CBC complex bind to caps on all mRNA and as such have a high specificity for the ^m7^G cap, but little preference for the identity of the first transcribed nucleotide (46,47). LARP1 is a specialised cap binding protein which has a >90 fold preference for TOP RNA (in which C is the first transcribed nucleotide) over mutated TOP RNA with G as the first transcribed nucleotide (40). Therefore, we initially focussed on investigating a potential role for LARP1 in mediating the impact of RNMT on TOP-RNA expression, although other cap binding proteins are also likely to have a role in regulating their stability, export and expression (see later). Importantly, other established TOP RNAs were repressed in *Rnmt* cKO CD4 T cells (Figure 3A), and RP and other TOP RNAs were repressed in RNMT-RAM KD HeLa cells (Supplementary Figure S2G), and in RNMT-compromised U2OS cells, indicating a conserved mechanism linking TOP RNA abundance to RNMT activity (16,48).

### LARP1 binding is predictive of RNMT dependence

LARP1 has multiple modes of RNA binding. The LA-module, made up of the LA-motif, PAM2 (PABPC1-binding) domain and RRM (RNA recognition motif), can bind to the TOP motif in a cap-independent manner (49). The LA-module also recognises the polyA tail directly and through interacting with the polyA-binding protein PABPC1 (23,24,44,49,50). The DM15 domain is the cap-binding domain and binds the cap structure and TOP motif (40,51,52). The DM15 domain binds to the RNA cap through a pi-stacking mechanism consistent with a preference for the more positively charged ^m7^G-cap over a G-capped structure (40,52). Accordingly, we found that LARP1 had increased interaction with ^m7^GTP over GTP *in vitro* (Figure 3D).

In HEK293 cells, PAR-CLIP analysis demonstrated that LARP1 is bound predominantly to the CDS and 3′UTRs (39). We sought to identify candidates for cap-dependent (5′ proximal) LARP1 binding in naïve CD4 T cells by enhanced crosslinking immunoprecipitation (eCLIP) sequencing analysis (Supplementary Figure S3A) (32). In contrast to the previous report, we found that in CD4 T cells, the majority of direct LARP1-RNA interactions occurred close to transcript start sites (TSS) in both coding (Figure 3E) and non-coding (lnc-RNA and pseudogenes) targets (Figure 3F). The TSS-adjacent binding pattern was observed with three anti-LARP1 antibodies (Figure 3G). Overall, we identified 367 LARP1 binding sites within transcript 5′ UTRs, and these had the expected pyrimidine rich motif (Supplementary Figure S3B, Figure 3H, Supplementary Table S3). These TSS-adjacent and TOP motif-containing binding sites are good candidates for cap-dependent binding through the DM15 domain. Several binding sites were also identified in long non-coding RNA (lncRNA), including snoRNA host genes, and upstream of or within RP and translation factor pseudogenes (Supplementary Figure S3B) (53). Examples of LARP1 eCLIP data alignment to individual coding and non-coding genes are shown in Figure 3I and Supplementary Figure S3C. CAGE data from the FANTOM5 project are shown as a reference (54).

**Figure 4.**
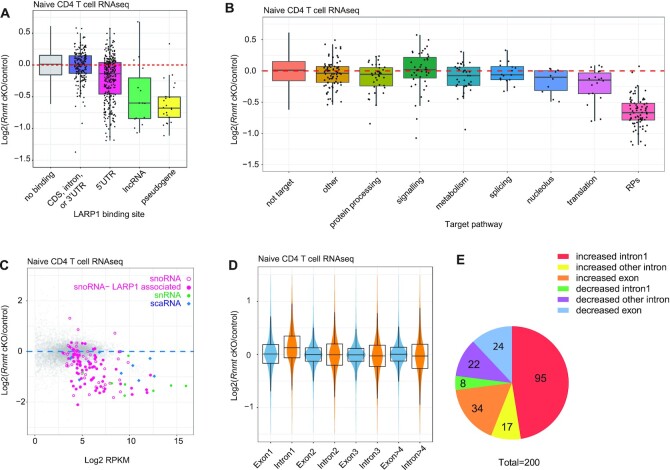
Small RNAs are sensitive to loss of *Rnmt* (A, B) Fold change in RNA expression from *Rnmt* cKO vs control naïve T cell RNAseq analysis. LARP1 target transcripts identified by eCLIP. (**A**) Transcripts grouped by LARP1 binding position and transcript biotype. (**B**) Transcripts with LARP1 binding to the 5′UTR grouped by functional pathway. (**C**) sRNA expression in RNAseq analysis of control (*n* = 3) and *Rnmt* cKO (*n* = 3) naïve CD4 T cells. sRNAs are grouped by RFAM family and highlighted if at least one gene overlaps with a LARP1 target, defined as 5′UTR or non-coding RNA binding. (D, E) Splicing analysis performed using RNAseq data from control (*n* = 3) and *Rnmt* cKO (*n* = 3) naïve CD4 T cells. Exons and introns reads normalised to total reads for that transcript. Then exon and intron read densities for each transcript compared between controls and *Rnmt* cKOs. (**D**) Violins represent the frequency density. Box plots show median, upper and lower quartiles. Whiskers, 1.5× interquartile range. (**E**) Pie chart of significantly altered introns and exons determined using DEXseq. Numbers indicate the number of significant differential splicing events in each group.

LARP1 binding to 5′UTRs, but not other sites, correlated with mRNA repression in the *Rnmt* cKO CD4 T cells (Figure 4A). LARP1-bound lncRNA (or snoRNAs derived from them) and pseudogenes were also repressed in *Rnmt* cKO cells (Figure 4A). mRNAs identified as LARP1 targets included the classical TOP transcripts encoding RPs and translation factors and additional targets in other pathways including signalling, energy metabolism, splicing and nucleolar functions; amongst these functional groups, RPs, translation factors and nucleolar proteins were most consistently affected by *Rnmt* loss (Supplementary Table S3, Figure 4B). Since LARP1 can bind to the TOP motif via the La-module in a cap-independent manner, it is likely that some of the RNMT-insensitive LARP1 targets are bound through this mechanism, which may have distinct regulatory effects (49).

The pattern of LARP1 binding in *Rnmt* cKO cells was similar to controls (Figure 3E, F, I, Supplementary Figure S3C), and notably the expression of LARP1 itself is not altered in *Rnmt* cKO CD4 T cells (Supplementary Figure S4A, B). As discussed earlier, only fully methylated ^m7^GpppC_m_, and not GpppC_m_, was readily detected in *Rnmt* cKO CD4 T cells, whereas unmethylated caps on non-TOP RNAs, GpppA_m,_ Gppp^m6^A_m_ and GpppG_m,_ were abundant (Figure 2F, G). These data imply that only TOP-RNAs with a mature cap (i.e. ^m7^GpppC_m_) are stable, and GpppC_m_ TOP mRNA is degraded leading to the selective loss of GpppC_m_ transcripts in *Rnmt* cKO CD4 T cells. TOP RNAs with incomplete cap structures would be predicted to bind less efficiently to LARP1, which could be responsible for their reduced stability in *Rnmt* cKO cells. In addition, we cannot rule out the enhanced dependency of TOP RNAs on other cap binding proteins or associated factors.

### Expression of small RNAs and mRNA processing is RNMT-dependent

Many small RNAs including snRNAs, scaRNAs, snoRNAs and their precursors are initially synthesised with a ^m7^G cap. In addition, several snoRNAs are excised from the introns of pre-mRNAs or lncRNA hosts, of which we identified 60 as LARP1-bound (Supplementary Table S3). 50 snoRNAs were detected in the naive CD4 T cell RNAseq analysis, of which 33 were reduced in *Rnmt* cKO T cells (Figure 4C, Supplementary Table S4), these included both those encoded within LARP1 targets and those that were not known to be associated with LARP1. All RNAPII-transcribed snRNAs were repressed in naïve *Rnmt* cKO CD4 T cells compared to controls (Figure 4C, Supplementary Table S4). The snRNA U6 was not repressed, as expected, since as a RNAPIII-transcript it is synthesised without a ^m7^G cap. Four of the five scaRNAs detected were also repressed in the *Rnmt* cKO T cells compared to controls. Interestingly, the expression of several snoRNA and snRNAs were also reduced in RNMT-RAM KD HeLa cells, indicating a conserved role for RNMT in small RNA expression (Supplementary Figure S4C) (16).

snoRNAs are involved in the cleavage, methylation and pseudouridylation of ribosomal RNA, and are required for ribosome biogenesis (see later). snRNAs are essential constituents of spliceosomes and scaRNAs guide snRNA modifications. Additionally, a major function of the RNA cap is to promote splicing of the first intron via an interaction with cap binding complex (CBC) (55–57). Therefore, we investigated splicing in naïve CD4 T cells. *Rnmt* cKO T cells had a mild defect in first intron splicing resulting in a relative increase in reads aligning to intron 1 across many genes (Figure 4D-E). This finding was recapitulated in cytoplasmic RNA from activated T cells (Supplementary Figure S4D-E). The intron 1 bias of the splicing defect in *Rnmt* cKOs is consistent with defects in CBC recruitment rather than a general effect on splicing. Following activation, *Rnmt* cKO CD4 T cells have less mRNA per cell than their control counterparts (Figure 2A); reduced mRNA processing linked to loss of CBC interaction is likely to contribute towards this effect.

### RNMT is essential for T cell activation

**Figure 5.**
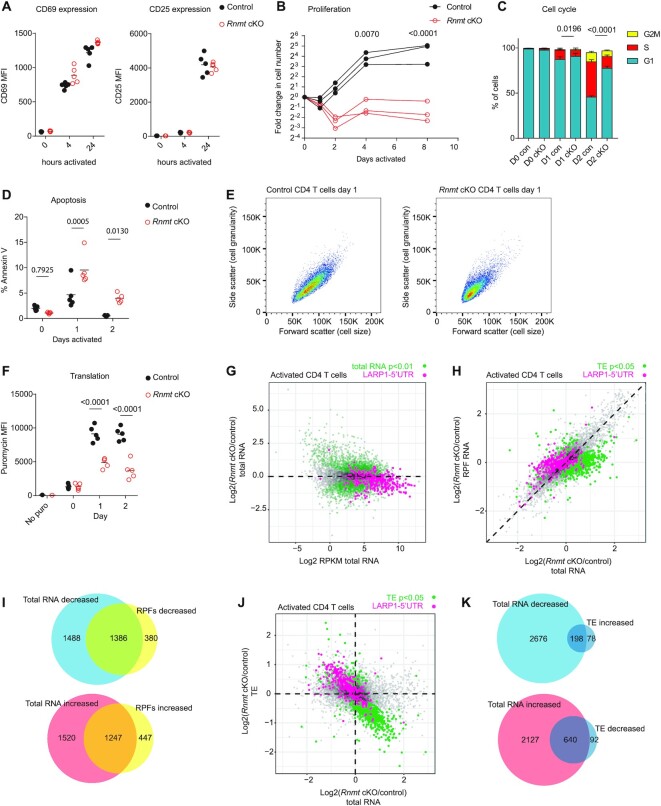
*Rnmt* cKO T cells have a defect in protein synthesis following activation (A–F) Control and *Rnmt* cKO lymph node cells were activated, after 2 days IL2 was added. (**A**) FACS analysis of CD69 and CD25 in control (*n* = 5) and *Rnmt* cKO (*n* = 5) CD4 T cells. Median fluorescence intensity (MFI) shown. On dot plots, dots indicate biological replicates, line indicates mean. (**B**) Proliferation of control (*n* = 3) and *Rnmt* cKO (*n* = 3) CD4 T cells. (**C**) FACS analysis of cell cycle of control (*n* = 5) and *Rnmt* cKO (*n* = 5) CD4 T cells, *P*-values are for % S phase cells. Bar indicates the mean, error bars indicate standard deviation. (**D**) Percent of control (n = 5) and *Rnmt* cKO (*n* = 5) annexin V+ CD4 T cells. (**E**) Example FACS plots showing forward and side scatter of control (1 of *n* = 5) and *Rnmt* cKO (1 of *n* = 5) CD4 T cells following one day of activation. (**F**) MFI of Puromycin incorporation into nascent peptides in control (*n* = 5) and *Rnmt* cKO (*n* = 5) CD4 T cells. *P*-values from ANOVA tests with Sidak's post test. (G–K) Ribosome footprinting analysis of cytoplasmic RNA from control (*n* = 3) and *Rnmt* cKO (*n* = 3) 20 h activated CD4 T cells. On scatter plots dots represent genes. Genes with LARP1 binding to transcript 5′UTR in pink. (**G**) MA plot of total RNA expression. Reads per million mapped reads (RPKM) on x-axis. Control and *Rnmt* cKO samples compared using EdgeR Exact Test. (**H**) Comparison of fold changes in total RNA and ribosome protected fragment (RPF) RNA. Differential translation efficiency (TE) in control and *Rnmt* cKO was calculated using Ribodiff. (**I**) Venn diagram displaying overlap between differentially expressed total RNA and RPF RNA, both *P* < 0.05 with EdgeR exact test. (**J**) Comparison of fold changes in total RNA and translation efficiency (TE). (**K**) Venn diagram displaying overlap between differentially expressed total RNA *P* < 0.05 with EdgeR exact test, and translation efficiency, *P* < 0.05 with Ribodiff. Figures representative of (A– F) two experiments.

Having established that RNMT has gene-specific impacts in naïve T cells, we proceeded to determine its role in T cell activation where transcription, translation and RNMT activity are considerably increased (Figure 1B,C). *Rnmt* cKO and control lymph node cells were activated *ex vivo* for 2 days by CD3 and CD28 co-receptor stimulation, followed by IL2 addition. The high affinity IL2 receptor, CD25, and type C lectin, CD69, hallmarks of TCR stimulation, were induced equivalently in *Rnmt* cKO and control CD4 and CD8 T cells following activation, indicating that TCR signalling is intact and induction of certain proteins is RNMT-independent (58) (Figure 5A, Supplementary Figure S5A). Control T cells proliferated rapidly following stimulation whereas *Rnmt* cKO T cells failed to proliferate, had delayed cell cycle entry after activation, and exhibited increased apoptosis (Figure 5B–D, Supplementary Figure S5B, C). Activated *Rnmt* cKO T cells had lower forward scatter, a proxy of cell size, than controls (Figure 5E, Supplementary Figure S5D). Puromycin incorporation, an indicator of the number of translating ribosomes, was decreased in *Rnmt* cKO T cells following activation (27) (Figure 5F, Supplementary Figure S5E). Thus, the biosynthetic pathways driving increased protein synthesis, cell growth, and cell cycle entry are RNMT-dependent.

### RNMT has a gene-specific impact on transcript abundance in activated T cells

We determined the transcriptome-wide effects of *Rnmt* deletion in CD4 T cells following 24 h of activation: of 10,126 genes analysed, 2874 were significantly repressed and 2726 were significantly increased (Figure 5G, Supplementary Table S5). In activated CD4 T cells, the mRNA per cell was reduced in *Rnmt* cKOs by 30% compared to controls, reflecting the impact of RNMT on mRNA transcription, processing and stability (Figure 2B). Because of this impact on RNA levels, single gene repression will be underestimated and upregulation overestimated in RNA seq analysis. LARP1 target mRNAs were repressed in *Rnmt* cKO activated CD4 T cells, and pathways analysis revealed decreased expression of mRNAs encoding RPs and proteins involved in fatty acid oxidation and synthesis, the electron transport chain and TCA cycle, and DNA repair and replication (Figure 5G, Supplementary Figure S5F). Decreased expression of genes involved in these biosynthetic and energy producing pathways is consistent with the decreased proliferation and delayed cell cycle entry observed.

Activated *Rnmt* cKO CD4 T cells had fewer translating ribosomes (Figure 5F), and a proportion of their mRNA lacked the ^m7^G cap required for efficient translation (Figure 2F, G). Thus, we investigated if there was a gene-specific reduction in translation. Ribosome footprinting detects ribosome-bound mRNA (ribosome protected fragments, RPFs), the relative abundance of which approximates translation efficiency (TE) (59). Since the number of translating mRNAs and total mRNA abundance are lower in *Rnmt* cKOs most mRNA will be translationally repressed compared to controls. Therefore, we cannot infer absolute differences in translation, but can compare relative changes in efficiency of translation, i.e. whether certain mRNAs are preferentially translated in *Rnmt* cKOs or whether all are equally repressed. For the majority of mRNAs, the fold change in RPFs matched the fold change in mRNA, indicating equivalent TE in *Rnmt* cKOs compared to controls (Figure 5H-I). However, in the *Rnmt* cKO, 732 mRNAs had a significantly decreased TE, of which the majority had increased total RNA; and 276 mRNAs had a significantly increased TE, of which the majority had decreased total RNA (Figure 5J, K, Supplementary Table S5). Despite the loss of RNMT activity (Figure 1E, F), much of the mRNA in *Rnmt* cKO CD4 T cells had a ^m7^G cap (Figure 2F, G), however, the percentage ^m7^G-capped RNA varied depending on the first transcribed nucleotide. In the *Rnmt* cKO cells, RNAs initiating with C, which includes TOP-RNAs, had the greatest proportion of caps with a ^m7^G structure (Figure 2F, G). We would expect mRNAs with the ^m7^G cap to have greater translation efficiency due to their greater affinity to the translation initiation factor EIF4E (46). Accordingly, of the 276 mRNAs with increased TE in *Rnmt* cKO, 93 were LARP1 target (C-capped) mRNAs. Conversely, we predict that those RNAs that are stable in the absence of a ^m7^G cap will be translationally repressed mRNA in the *Rnmt* cKO.

### Ribosome protein synthesis is RNMT-dependent in activated CD4 T cells

**Figure 6.**
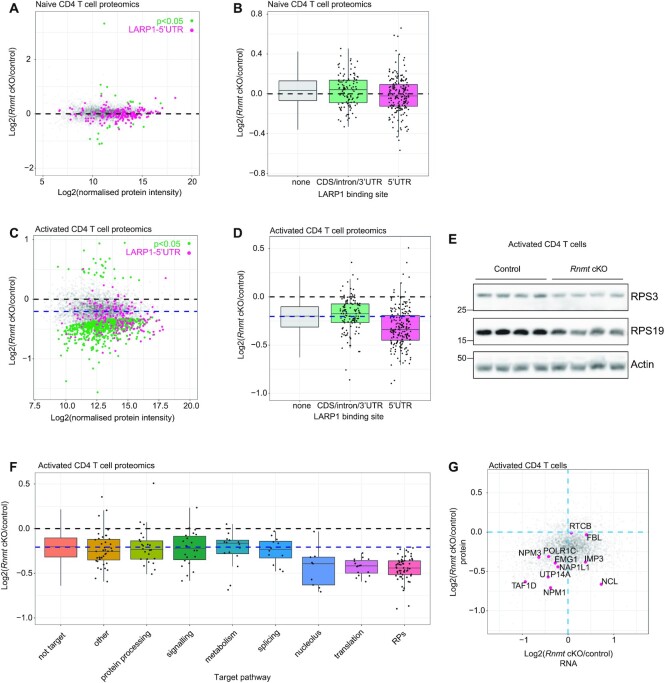
RNMT promotes expression of ribosome biogenesis factors in activated T cells TMT proteomics analysis of (**A, B**) control (*n* = 4) and *Rnmt* cKO (*n* = 3) naïve CD4 T cells; (C, D) control (*n* = 4) and *Rnmt* cKO (*n* = 4) 20 h-activated CD4 T cells. (A, C) MA plot of protein expression. Control and *Rnmt* cKO samples compared by linear modelling in Limma using histone protein intensities to normalise expression between samples. Black dashed line is at Log2 (*Rnmt* cKO/control) = 0 and blue dashed line on (**C**) indicates the median Log2(*Rnmt* cKO/control). (B, D) Fold change in proteins encoded by LARP1 target transcripts (eCLIP), in *Rnmt* cKO vs control naïve. Black dashed line is at log_2_ (*Rnmt* cKO/control) = 0 and blue dashed line on (**D**) indicates the median log_2_ (*Rnmt* cKO/control). (**E**) Western blot analysis of ribosomal proteins in control (*n* = 4) and *Rnmt* cKO (*n* = 4) 20 hour activated CD4 T cells. (**F**) Fold change in proteins in *Rnmt* cKO versus control activated CD4 T cells, proteins encoded by LARP1 target transcripts (eCLIP), defined here as 5′UTR binding, are grouped by pathway. Black dashed line is at log_2_ (*Rnmt* cKO/control) = 0 and blue dashed line indicates the median log_2_ (*Rnmt* cKO/control). (**G**) Comparison of protein and RNA expression from 20 h activated T cell cytoplasmic RNAseq and TMT proteomics analyses. Nucleolar proteins with LARP1 binding to their transcript 5′UTR mRNA highlighted.

We investigated whether deletion of *Rnmt* had an impact on the proteome of naïve and activated CD4 T cells using quantitative mass spectrometry. This revealed few differences in protein expression in *Rnmt* cKO naïve T cells compared to controls; of 3874 proteins detected 12 were significantly increased and 12 were significantly decreased (Figure 6A, Supplementary Table S6). Although LARP1 target mRNAs were repressed in these cells, their proteins were minimally affected (Figures 3A and 6A, B). TOP mRNA is known to be translationally repressed in naïve T cells, and thus forms a pre-transcribed pool poised for expression upon T cell activation (3). Indeed, following activation, many proteins were expressed in a *Rnmt-*dependent manner (Figure 6C, D, Supplementary Table S7). Consistent with decreased protein synthesis and cell size, the protein content was about 15% lower in *Rnmt* cKO cells than controls (Figure 6C, D). From 4495 proteins detected, 838 proteins were significantly decreased and 32 were increased. Pathway analysis indicated that RPs were the most strongly repressed proteins, with translation factors, DNA replication and cholesterol biosynthesis also repressed (Supplementary Figure S6A). Reduced expression of the RPs RPS3 and RPS19 in the *Rnmt* cKO activated CD4 T cells was confirmed (Figure 6E, Supplementary Figure S6B).

In activated *Rnmt* cKO CD4 T cells, we found a correlation between LARP1-binding to the transcript 5′UTR and repression of the protein product (Figure 6D). Thus, although in naïve RNMT cKO T cells the selective repression of TOP RNAs had minimal impact on protein synthesis, these transcripts were critical for protein expression following activation. Of LARP1 target transcripts groups, the most repressed proteins were RPs, translation factors and nucleolar proteins (Figure 6F). Focussing on the nucleolar proteins which contribute to ribosome biogenesis (Figure 6G), the *Rnmt* cKO activated T cells had reduced expression of the RNAPI component TAF1D, RNAPI/III component POL1RC and nucleolin (NCL) all of which promote rRNA transcription (60–62). Components of the U3 snoRNP which mediates rRNA cleavage, UTP14a and IMP3 (63,64), the pseudouridine methyltransferase EMG1 and nucleophosmins 1 and 3 were also decreased (65,66). These ribosome biogenesis factors all have LARP1 binding sites.

In HeLa cells, RNMT regulates ribosome biogenesis through upregulation of *c-MYC* (67). Notably c-Myc mRNA was induced rather than repressed on *Rnmt* deletion in activated CD4 T cells and there was no evidence of repression of the Myc transcriptional program (Supplementary Figure S6C). Conversely RNMT expression is dependent on Myc in activated T cells and induction of RNMT is likely to contribute to the mechanism through which Myc induces ribosome biogenesis (Supplementary Figure S6D) (68).

### Ribosome biogenesis is RNMT-dependent in activated CD4 T cells

**Figure 7.**
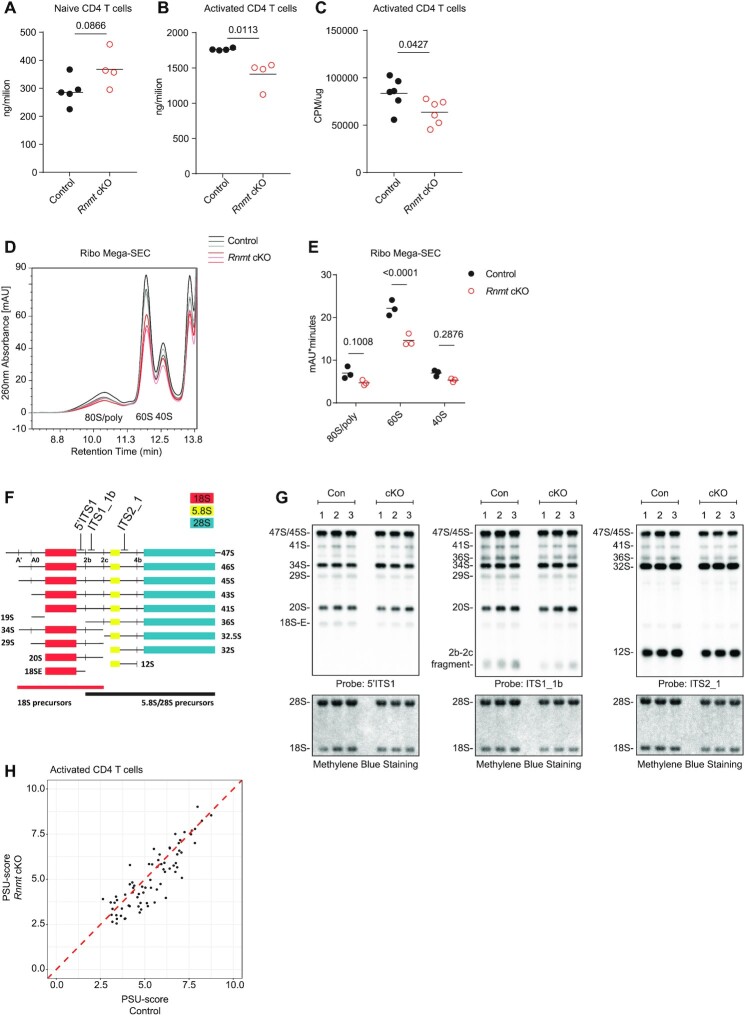
RNMT promotes ribosome biogenesis in activated T cells (**A**) RNA/cell from control (*n* = 5) and *Rnmt* cKO (*n* = 4) naïve CD4 T cells. Dots represent biological replicates, bars indicate means. P values from Student's *t*-test. (B, C) 20 h activated CD4 T cells were incubated with ^3^H uridine for 4 h. (**B**) RNA per million control (*n* = 4) and *Rnmt* cKO (*n* = 4) cells. (**C**) CPM per μg RNA from control (*n* = 6) and *Rnmt* cKO (*n* = 6) cells (data combined from two experiments). Dots represent biological replicates, bars indicate mean. *P* values from Student's *t*-test. (D, E) Ribo Mega-SEC analysis of control (*n* = 3) and *Rnmt* cKO (*n* = 3) activated CD4 T cells, equivalent cells were loaded. (**D**) Polysome profiles. Lines represent biological replicates. (**E**) Peak areas for 80S/polysomes, 60S and 40S ribosomes. Dots indicate biological replicates. Line indicates the mean. *P* values from an ANOVA with Sidak's post-test. (F, G) Northern blot analysis of rRNA intermediates in control (*n* = 3) and *Rnmt* cKO (*n* = 3) activated CD4 T cells. (**F**) Diagram explaining the fragments and probes. (**G**) Northern blots. Each column number represents a biological replicate. (**H**) Pseudouridine (Ψ)-seq analysis of control (*n* = 4) and *Rnmt* cKO (*n* = 4) activated CD4 T cells. PSU-score = (read starts/read coverage) CMC treated/(read starts/read coverage) mock treated for nucleotide downstream of Ψ. Samples compared by linear modelling in Limma, no significant changes found. Figures representative of (D, E) three experiments.

Since the ribosomal proteins, snoRNAs and ribosome biogenesis factors were repressed following *Rnmt* deletion, we investigated the impact on ribosome biogenesis. Whilst the total RNA (mostly rRNA) content of naïve CD4 T cells was unchanged on *Rnmt* deletion, following activation the RNA per cell and net rate of total RNA synthesis/decay was reduced in *Rnmt* cKO T cells compared to controls (Figure 7A-C). Activated *Rnmt* cKO CD4 T cells had lower levels of monosomes and polysomes than controls, as assessed by Ribo Mega-SEC profiling (29) (Figure 7D, E). Thus, RNMT is critical for the induction of ribosome biogenesis following T cell activation.

Having established that ribosome biogenesis was reduced in *Rnmt* cKOs we investigated the impact on ribosome quality control. We characterised the protein content of ribosomes in activated *Rnmt* cKO CD4 T cell ribosomal fractions and found no significant differences in relative abundance of any RP in any ribosomal fraction (Supplementary Figure S7, Table S8). snoRNAs can either guide pre-rRNA cleavage, pseuouridylation or ribose 2′O-methlyation and we found examples of each class to be downregulated in *Rnmt* cKO CD4 T cells (Supplementary Table S4). Analysis of rRNA processing intermediates demonstrated equivalent rRNA processing in the control and *Rnmt* cKO activated CD4 T cells (Figure 7F, G). Pseudouridine (Ψ)-seq analysis of rRNA activated CD4 T cells detected pseudouridine in 75 positions (33), but revealed no differences in the conversion of uridine to pseudouridine in the control and *Rnmt* cKO (Figure 7H, Supplementary Table S9). Thus, RNMT controls the rate, but not the quality of ribosome biogenesis.

In conclusion, we report that during T cell activation, upregulation of RNMT co-ordinates mRNA capping and maturation with increased translational capacity by selectively upregulating the proteins and non-coding RNAs involved in ribosome biogenesis.

## DISCUSSION

When T cells are activated by interaction with a cognate antigen, the transition from naïve to activated phenotype is driven by the induction of biosynthetic pathways. Here, we identified the ^m7^G cap methyltransferase RNMT as a key mediator of T cell activation, induced by TCR stimulation. The ^m7^G cap has previously defined biochemical roles in RNA processing and translation, however, prior to this study its influence on endogenous cellular transcripts in mammals had been unclear because the ^m7^G capping enzymes, RNGTT and RNMT, are essential for cell viability in most lineages (69).

An important observation was that although cap guanosine N-7 methyltransferase activity was undetectable on *Rnmt* deletion, ∼80% mRNA had a complete ^m7^GpppN_m_ cap in *Rnmt* cKO T cells. Although by all indicators *Rnmt* gene deletion was successful, residual RNMT protein may be sufficient to methylate a proportion of mRNA caps aided by recruitment of RNMT to sites of transcription; these transcripts would be more stable and better processed compared to RNA with incomplete cap structures (16,70). *Rnmt* cKO T cells, however, cannot induce RNMT protein upon activation where the accelerated production of nascent RNA transcription is likely to outstrip the cap methylation capacity of pre-existing, residual RNMT protein. An alternative explanation for the presence of the ^m7^GpppN_m_ cap in *Rnmt* cKO T cells is that there is a partially redundant ^m7^G methyltransferase, although such enzymes or activities have not been identified.

Although the ^m7^G cap is ubiquitous on endogenous cellular RNAPII-transcribed RNA, a key finding here is that the expression of specific RNAs have enhanced dependency on RNMT, including TOP RNAs. The TOP RNAs are protected from degradation by interaction with a specialised ^m7^G-cap binding protein, LARP1. In *Rnmt* cKO CD4 T cells, the reduction in TOP transcripts and the reduction in ^m7^GpppC_m_ capped-RNA (predominantly TOP transcripts) are proportionate (∼30%), and incompletely methylated GpppC_m_ structures are rare compared to other incomplete structures such as GpppG_m_, implying that GpppC_m_-capped RNA is unstable. Thus, the protective relationship between TOP RNAs and the cap binding protein LARP1 may selectively sensitise them to RNMT loss.

The link between RNMT expression and TOP-RNA expression is robust, being present in naïve and activated mouse CD4 T cells as well as human cancer cells. Despite this strong correlation between LARP1-RNA binding and RNMT-dependency, other mechanisms could also contribute to the specification of RNMT-dependent RNAs. For instance, TOP-RNAs may be more dependent on other cap binding complexes such as CBC, eIF4E and export complexes for their splicing, co-transcriptional stability or export, all of which can contribute to RNA stability. However, unlike LARP1, other analysed cap binding proteins have no significant binding preference towards the caps of TOP RNAs over other ^m7^G-capped RNAs. It is also possible that other factors influence RNMT-dependent expression of TOP and other RNAs, such as accessibility of RNA to RNMT or speed of transcription. Although we have focussed mainly on the RNMT sensitive transcripts in this study it would also be informative to investigate why many transcripts are RNMT-insensitive.

TOP RNAs are translationally repressed in naïve T cells, poised to be expressed in response to activation, permitting rapid transition to the activated phenotype by accelerating ribosome biogenesis (3). In unstimulated naïve T cells, although loss of *Rnmt* affects the transcriptome by altering the expression of the translationally repressed TOP RNAs there is little impact on the proteome. Upon activation, T cells increase their ribosome content, translational capacity, increase in size and undergo rapid proliferation. RNMT is required for the expression of TOP-encoded ribosomal proteins and biogenesis factors and in the absence of *Rnmt*, T cells fail to increase their translational capacity resulting in reduced size, delayed cell cycle entry and ultimately apoptosis.

Since the role of LARP1 had not been investigated in T cells previously, we performed eCLIP analysis of LARP1-RNA interactions in naïve CD4 T cells to identify LARP1 target RNAs and map their site of interaction. In T cells, the majority of LARP1 binding occurred close to the TSS in both coding and non-coding RNAs, consistent with cap-dependent interactions through the DM15 domain. LARP1 can associate with the polyA tail either directly or through PABPC1 (23,24,44,49,50); our data suggests that most contacts with the 3′ end of RNAs would be indirect in T cells, however, direct binding to the polyA tail could potentially still be important for the initiation of this interaction. Not all the LARP1 targets identified were repressed by loss of *Rnmt*. LARP1 can also bind to the TOP motif through the LA-module in a cap-independent manner (49), which may have distinct regulatory effects, or these transcripts may recruit other RNA binding proteins that stabilise them (71–73). Interestingly, we also detected LARP1 binding to RP and translation factor pseudogenes. Although under certain conditions, these pseudogenes could potentially act as LARP1-sponges and compete with the other TOP mRNAs for LARP1 binding, in naïve CD4 T cells the pseudogene expression and LARP1 eCLIP read counts were much lower than their protein-coding counterparts.

Since the ribosomal proteins (RPs) were the most repressed functional group in our proteomics analysis of activated *Rnmt* cKO CD4 T cells, we focussed on ribosome biogenesis for this study. Ribosomes and translation factors are the canonical targets of LARP1, however, there were also LARP1 targets identified in metabolic pathways, splicing, protein processing and cell signalling. Some of these targets are novel and some have been identified as TOP RNAs in previous studies (74–76), or as transcripts whose translation is sensitive to MTOR pathway inhibition (77), a feature of TOP-RNA regulation. Co-regulation of translation, metabolism and cell signalling may integrate these co-dependent processes. The best characterised regulator of LARP1 is MTOR, a serine/threonine kinase that integrates signals from external and internal stimuli such as mitogens and nutrient availability (22,78,79). MTOR phosphorylates LARP1 adjacent to the DM15 domain, reducing its cap-binding to allow more efficient translation of TOP RNAs (24,42,45). We propose that RNMT functions upstream of this mechanism since efficient cap methylation allows LARP1 to stabilise a pool of TOP mRNA ready to be translated upon mitogenic stimuli such as TCR signalling in T cells.

In addition to affecting TOP RNA expression, RNMT has roles in non-coding RNA expression and mRNA processing, specifically in splicing out the first intron, a CBC-dependent process (55–57). snRNAs were repressed in *Rnmt* cKO CD4 T cells, probably due to destabilisation of those that do not gain a complete tri-methylguanosine cap structure, since the un-capped snRNA U6 is unaffected. *Rnmt* cKO CD4 T cells also had reduced expression of several snoRNAs. Capped snoRNAs interact with CBC to recruit snoRNA biogenesis factors, indicating a potential mechanism for their repression in *Rnmt* cKOs (80,81). Intron-derived snoRNAs are not capped so do not interact directly with CBC, however, it is possible that they recruit snoRNA biogenesis factors through the CBC prior to splicing. We identified LARP1 binding sites in 10 lncRNAs and 50 mRNAs that host snoRNAs, and one snoRNA, Snord104. LARP1 binding could have an impact on snoRNA host stability or processing, but but changes were also observed in snoRNAs whose hosts were not identified as LARP1 targets.

To conclude, we discovered that RNMT has a fundamental role in upregulating the expression of a TOP RNA regulon, driving ribosome biogenesis during T cell activation. Ribosome biogenesis is critical for proliferation during T cell activation and has important roles in other cells including driving inappropriate cell proliferation in cancerous cells where we have also observed a link between RNMT and TOP RNAs (8,9,82). Through the selective dependence of TOP RNA and snoRNA on RNMT, ribosome biogenesis is coordinated with the generation of ^m7^G capped transcripts during T cell activation.

## DATA AVAILABILITY

RNAseq data including ribosome footprinting, eCLIP and Ψ-seq are available at NCBI GEO datasets using reference GSE160328. Proteomics datasets are available from proteomeXchange with the following accessions: naïve T cell data PXD023793, activated T cell data PXD023799, Ribo Mega-SEC PXD023832.

## Supplementary Material

gkab465_Supplemental_Files
