# Supplementary material for: Upregulation of RNA cap methyltransferase RNMT drives ribosome biogenesis during T cell activation
Source: Nucleic Acids Res. 2021 Jun 14;49(12):6722–38. doi: 10.1093/nar/gkab465 (PMC8266598; doi:10.1093/nar/gkab465)
Supplement: gkab465_Supplemental_Files [file gkab465_supplemental_files.zip › Supplemental-11-legends.pdf]

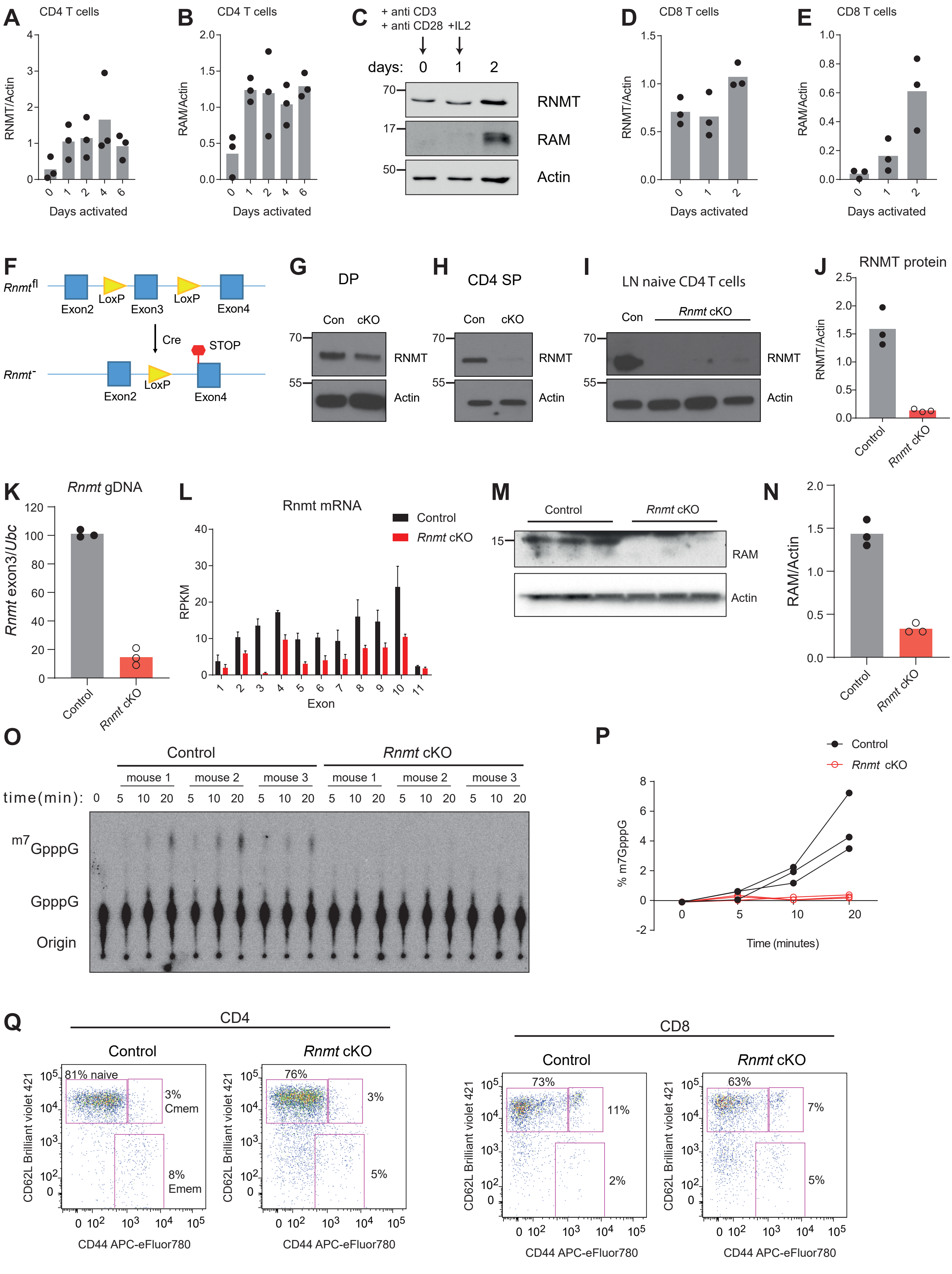

Supplemental fgure S1: Legend overleaf

Supplemental figure S1. ***Rnmt* cKO phenotype** **A-B)** Quantitation of 3 replicate western blots showing **A)** RNMT and **B)** RAM expression in activated CD4 T cells. **C-E)** Western blots of RNMT and RAM expression following CD8 T cell activation. **C)** Representative western blots, **D-E)** quantitation of 3 replicate western blots showing **D)** RNMT and **E)** RAM expression. **F)** Targeting strategy of *Rnmt*^{fl} allele. **G-I)** Western blots showing expression of RNMT in **G)** double positive (DP), **H)** CD4 single positive (SP) cells sorted from *Rnmt* cKO (n=1) and control (n=1) thymi, and **I)** naïve CD4 T cells from *Rnmt* cKO (n=3) and control (n=1) lymph nodes. **J)** Quantitation of replicate western blots showing RNMT expression in 20 hour activated control and *Rnmt* cKO CD4 T cells. **K)** Genomic DNA qPCR for *Rnmt* exon3 normalised to *Ubc* in naïve CD4 T cells from control (n=3) and *Rnmt* cKO (n=3) mice. **L)** Quantitation of reads across each exon of the Rnmt transcript from RNAseq of naïve CD4 T cells from control (n=3) and *Rnmt* cKO (n=3) mice. **M-N)** Western blots of RAM expression in 24 hour activated control (n=3) and *Rnmt* cKO (n=3) CD4 T cells. **M)** Western blot and **N)** quantitation of western blot, a second blot with 2 control and 2 *Rnmt* cKO samples gave similar results. **O-P)** Cap methyltransferase activity assay in 4.1 x 10^5 control (n=3) and *Rnmt* cKO (n=3) magnet sorted naïve CD4 T cells activated *ex vivo* for 24 hours. **O)** Radiograph and **P)** quantification are shown. **Q)** Proportions of lymph node CD4 and CD8 T cells in naïve, central memory (Cmem), and effector memory (Emem) populations in control (n=5) and *Rnmt* cKO (n=5) mice. Representative FACS plots shown. Population names on control CD4 plot.

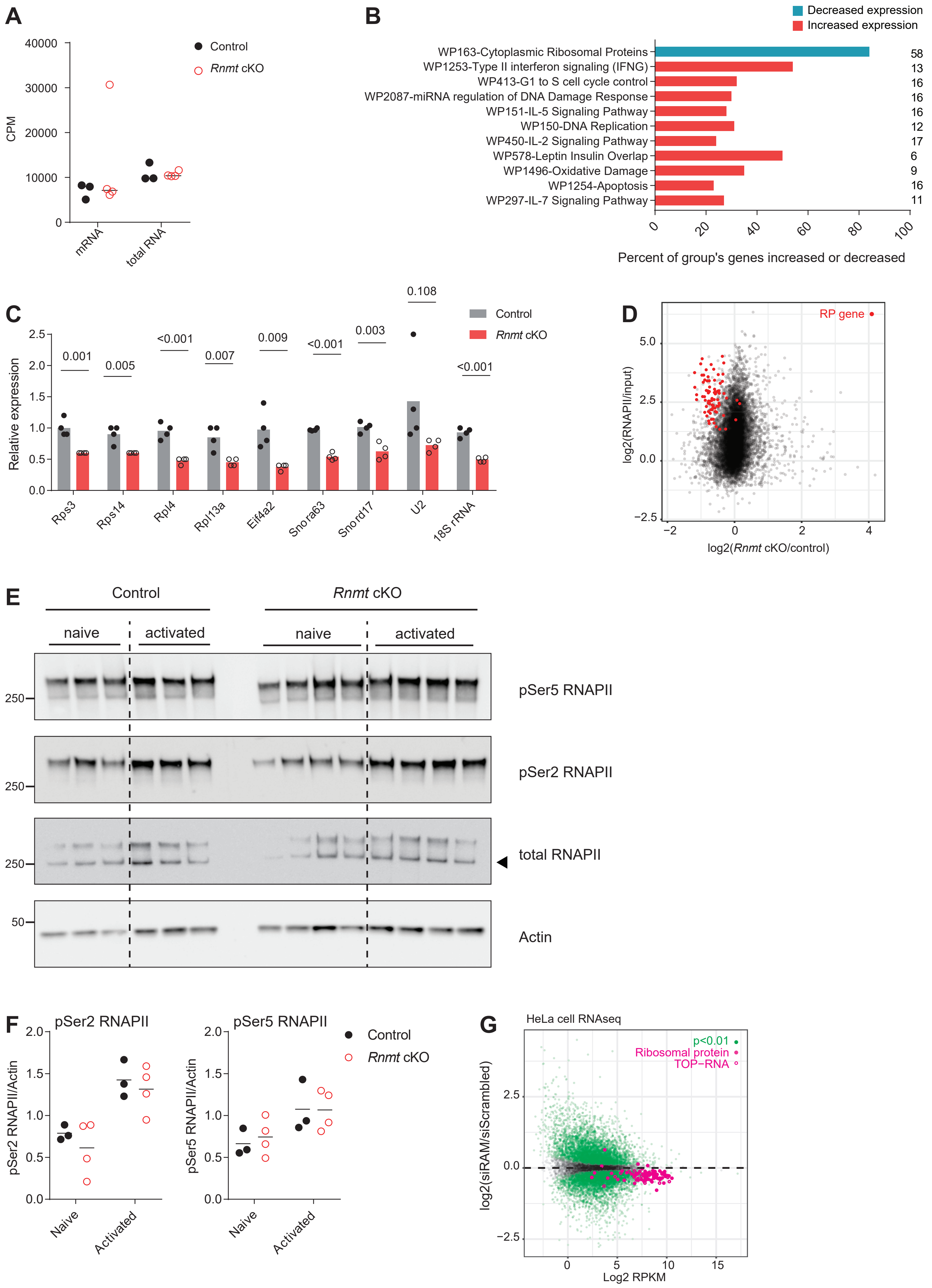

Supplemental figure S2: Legend overleaf

Supplemental figure S2. **Gene expression in *Rnmt* cKO CD4 T cells** **A)** ^{3}H signal in total and dT purified mRNA in control (n=3) and *Rnmt* cKO (n=4) 20 hour-activated CD4 T cells treated with ^{3}H methionine used for chromatography shown in Figure 2D-E. **B)** Pathway analysis of significantly changed genes (adjusted p value <0.05) from *Rnmt* cKO and control naïve CD4 T cell RNAseq. Pathways with FDR<30% selected, number of genes changed in each pathway indicated. **C)** RT-qPCR of selected RNMT-sensitive mRNAs in CD4 T cells from control (n=4) and *Rnmt* cKO (n=4) mice. Data was normalised to four control RNAs: Actb, Pten, Tbp and Ddx56 which were not differentially expressed in the RNAseq data. Control and *Rnmt* cKO samples were compared with t-tests using the Benjamini-Hochberg correction for multiple testing. **D)** *Rnmt* cKO (n=3) and control (n=3) naïve CD4 T cell RNAseq analysis data plotted against published RNAPII ChIP sequencing data on naïve CD4 T cells. **E)** Western blots of RNAPII expression and phosphorylation in 20 hour activated or naïve control (n=3) or *Rnmt* cKO (n=4) CD4 T cells. **F)** Quantitation of mean grey value of the western blots in **(E).** **G)** Published RNAseq analysis of HeLa cells treated with scrambled siRNA (n=5) and siRAM (n=5). MA plot of RNA expression, with RP and TOP RNAs highlighted.

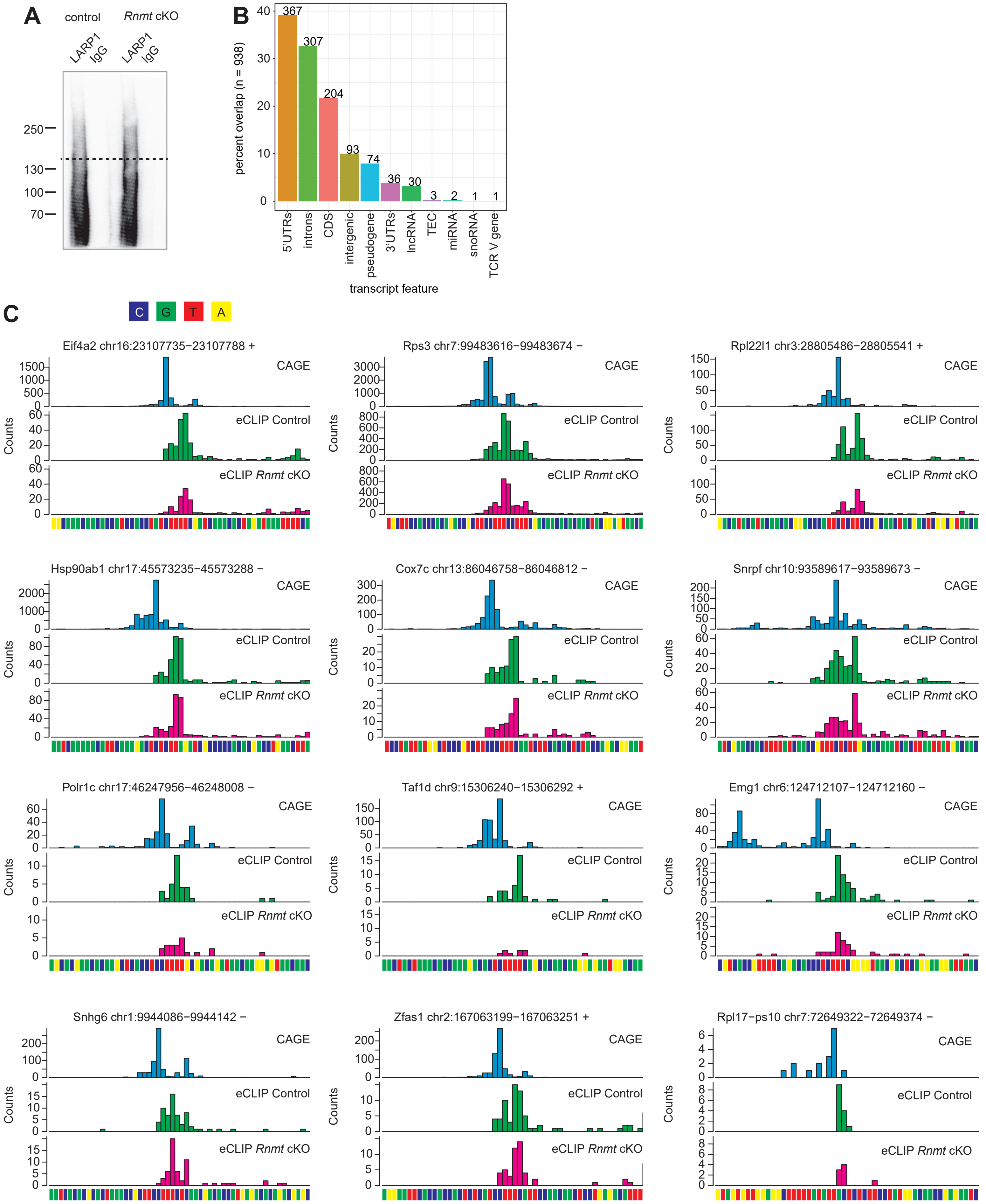

Supplemental figure S3. **LARP1 eCLIP A)** Detection of ^{32}P labelled RNA-protein complexes purified using anti-LARP1 or isotype control antibodies from control or *Rnmt* cKO naïve CD4 T cells. Unlabelled samples on a separate membrane were cut above the line to generate sequencing libraries. **B)** Transcript features in which LARP1 binding sites were identified. **C)** Control and *Rnmt* cKO LARP1 eCLIP reads aligning to example LARP1 target transcripts from a variety of pathways. CD4 T cell CAGE data from FANTOM 5 project shown for reference. Counts are the number of reads starting at that position. Sequence shown at bottom of each panel.

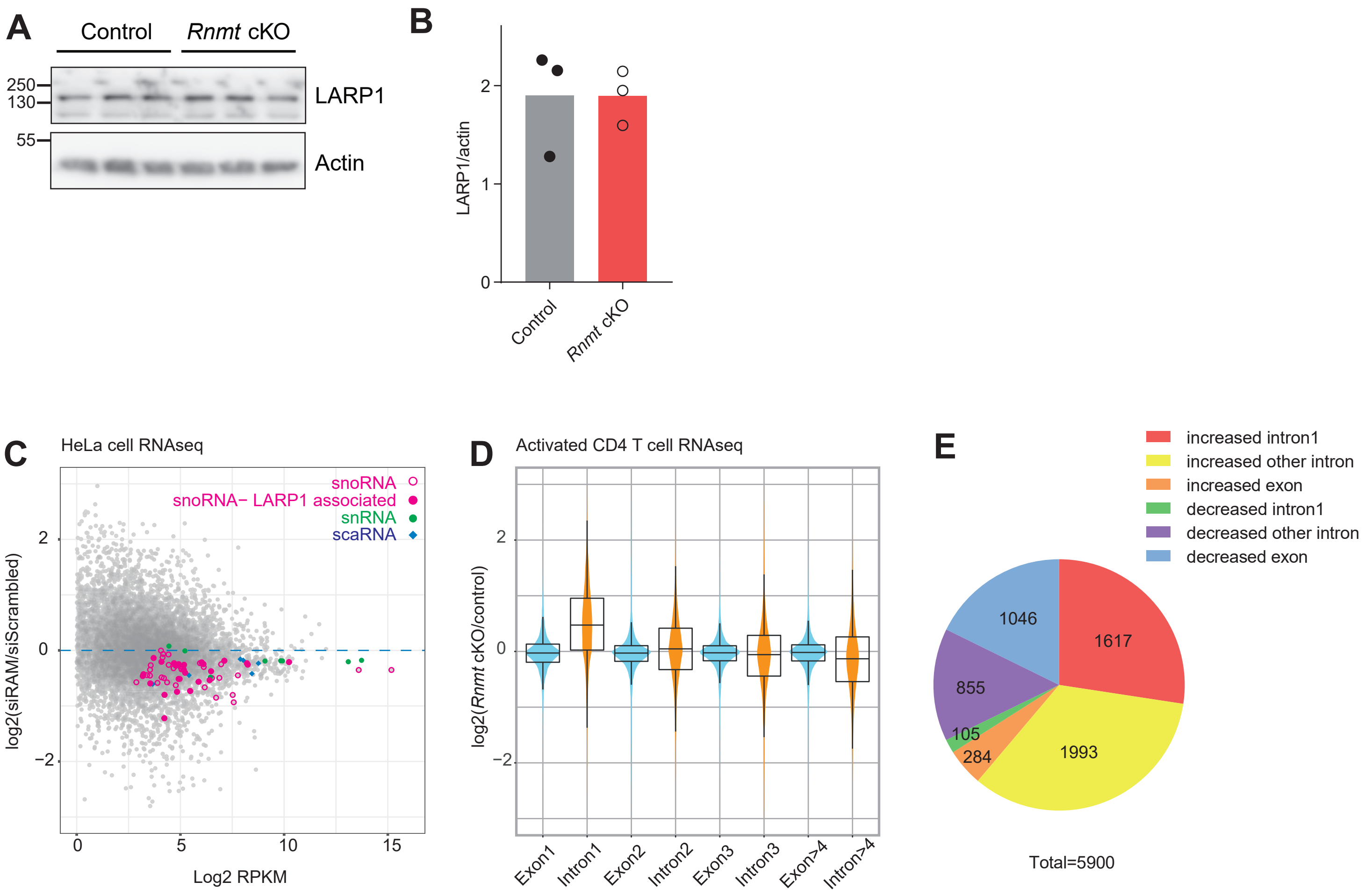

Supplemental figure S4. **Role for RNMT in non-coding RNA expression and splicing. A-B)** Western blot **(A)** and quantification **(B)** from western blotting detection of LARP1 in naïve CD4 T cells from control (n=3) and *Rnmt* cKO (n=3) mice. **C)** Reanalysis of published RNAseq data of HeLa cells treated with scrambled siRNA (n=5) and siRAM (n=5). MA plot highlighting expression of sRNAs: sRNAs are grouped by RFAM family and highlighted if at least one gene overlaps with a LARP1 target, defined as 5'UTR or non-coding RNA binding, in the mouse LARP1 dataset. **D-E)** Splicing analysis using RNAseq of ribosome depleted cytoplasmic RNA from control (n=3) and *Rnmt* cKO (n=3) activated CD4 T cells. Reads aligning to exons and introns normalised to the total reads for that transcript. Then exon and intron read densities for each transcript compared between controls and *Rnmt* cKOs. **D)** Violins represent the frequency density. Box plots show median, upper and lower quartiles. Whiskers, 1.5 X interquartile range. **E)** Pie chart of significantly altered introns and exons determined using DEXseq. Numbers indicate the number of significant differential splicing events in each group.

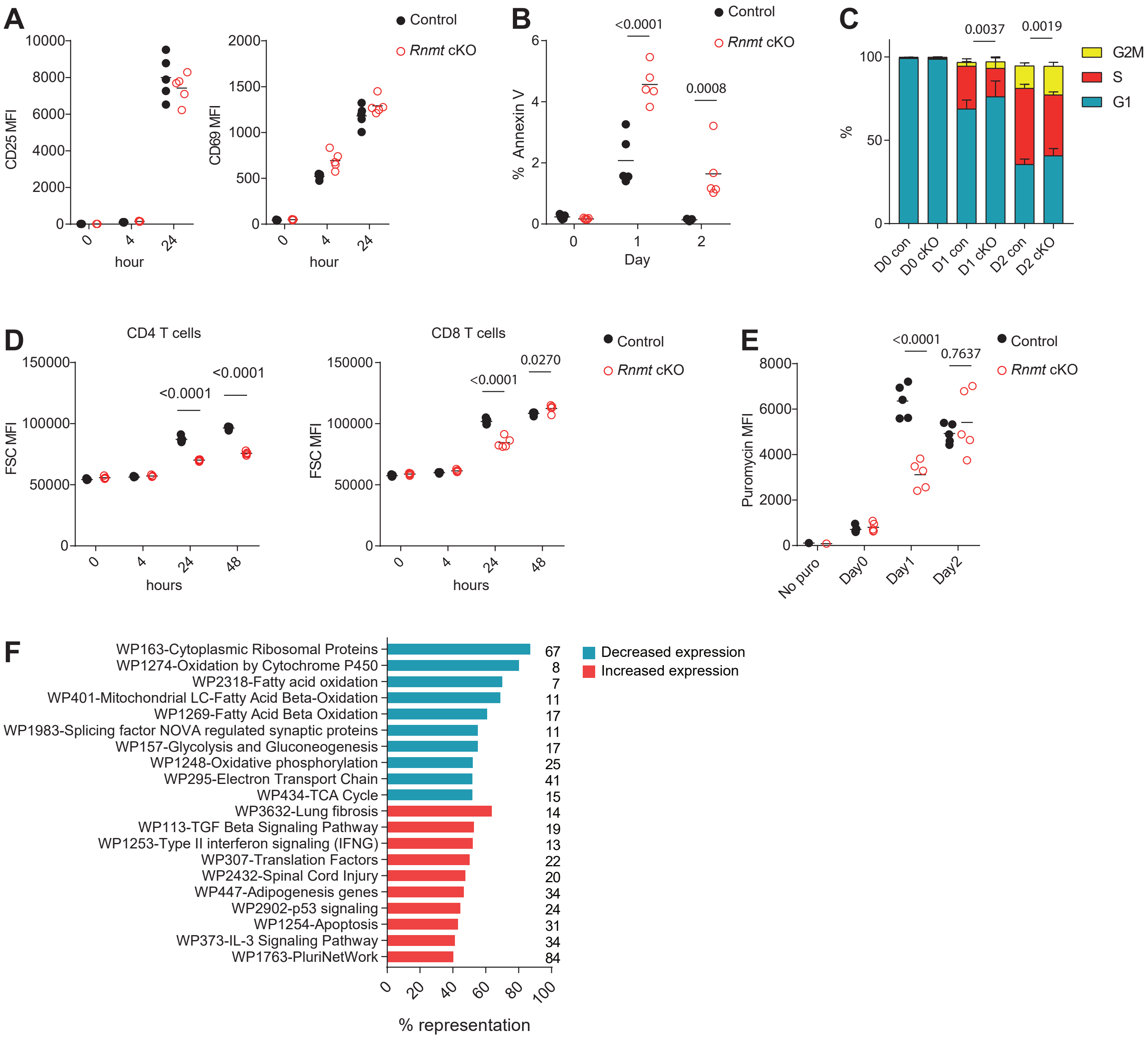

Supplemental figure S5. **Activation of *Rnmt* cKO T cells A-E)** Lymph node cells were activated and after two days IL2 was added. On dot plots each dot indicates a biological replicate, line indicates mean. P-values from ANOVA tests with Sidak's post test. **A)** Median fluorescence intensity (MFI) of CD25 and CD69 on control (n=5) and *Rnmt* cKO (n=5) CD8 T cells. **B)** Percent of control (n=5) and *Rnmt* cKO (n=5) annexin V+ CD8 T cells analysed by FACS. **C)** FACS analysis of cell cycle of control (n=5) and *Rnmt* cKO (n=5) CD8 T cells. P values are for % S phase cells. Bar indicates the mean, error bars indicate standard deviation. **D)** Forward scatter (FSC) MFI of control (n=5) and *Rnmt* cKO (n=5) CD4 and CD8 T cells. **E)** MFI of puromycin incorporation in control (n=5) and *Rnmt* cKO (n=5) CD8 T cells. **F)** Pathway analysis of significantly changed genes in control (n=3) and *Rnmt* cKO (n=3) activated CD4 T cell RNAseq analysis (adjusted p value<0.05). Pathways with FDR<30% selected, number of genes changed in each pathway indicated. Figures **A, C-E)** are representative of two experiments.

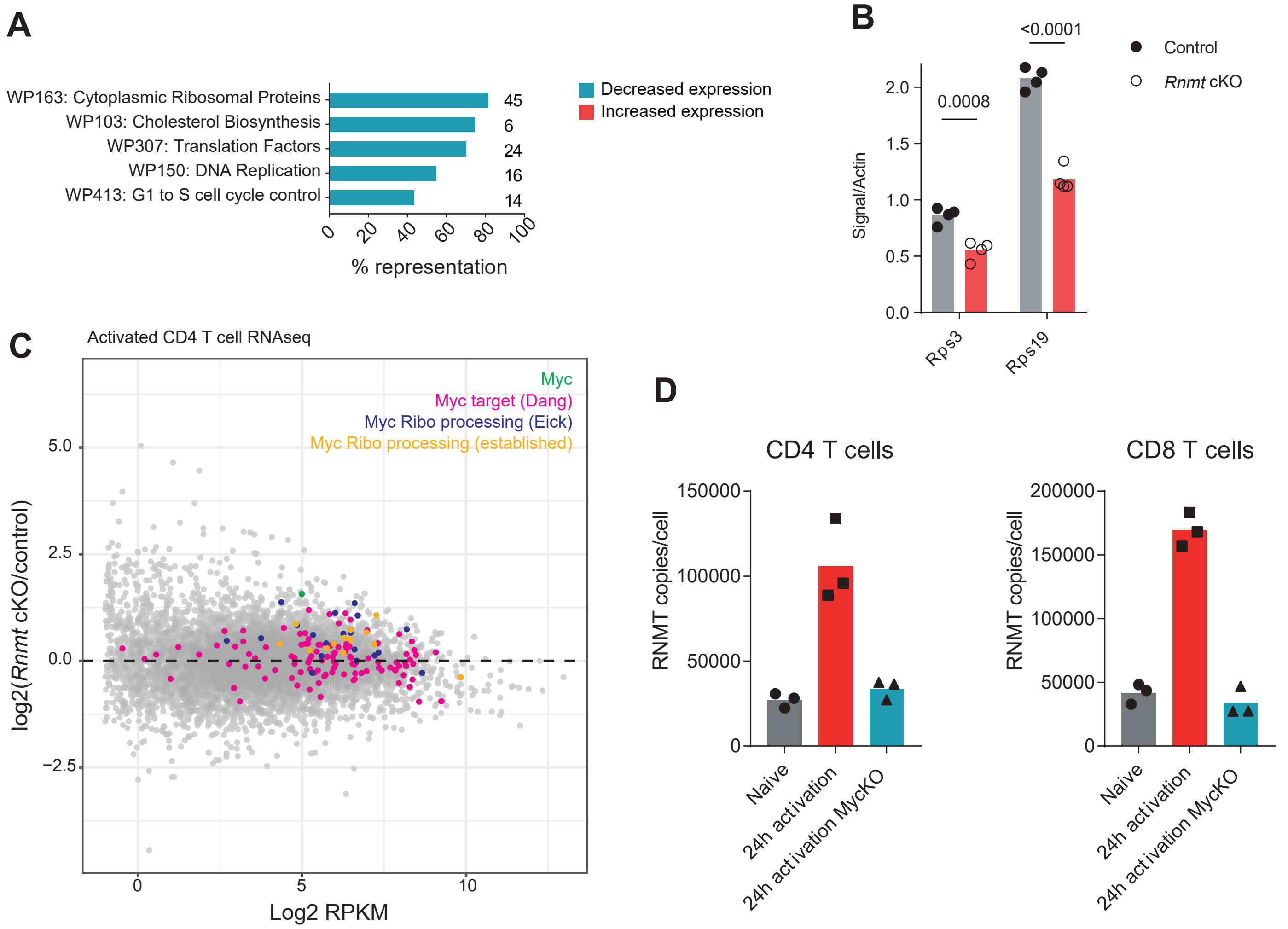

Supplemental figure S6. **RNMT promotes expression of ribosome biogenesis factors** **A)** TMT proteomics analysis of control (n=4) and *Rnmt* cKO (n=4) 20 hour-activated CD4 T cells. Gene pathway analysis of significantly changed proteins (adjusted p value<0.05). Pathways with FDR<30% selected. Number of genes changed in each pathway indicated. **B)** Quantitation of mean grey value of replicate western blots in Fig.6E showing RPS3 and RPS19 expression in activated control (n=4) and *Rnmt* cKO (n=4) CD4 T cells. Data were compared by an ANOVA with Sidak's post-test. **C)** RNAseq analysis of ribosome depleted cytoplasmic RNA from control (n=3) and *Rnmt* cKO (n=3) activated CD4 T cells. MA plot of RNA expression with *Myc* and MYC targets highlighted. "Myc target (Dang)" indicates genes from the Dang Myc target list in GSEA (Zeller, 2003). "Myc Ribo processing (Eick)" and "Myc Ribo processing (established)" indicate novel and previously reported Myc targets (Schlosser, 2003). **D)** Expression of RNMT protein in published proteomics datasets of activated CD4 and CD8 T cells from control and *c-Myc* KO

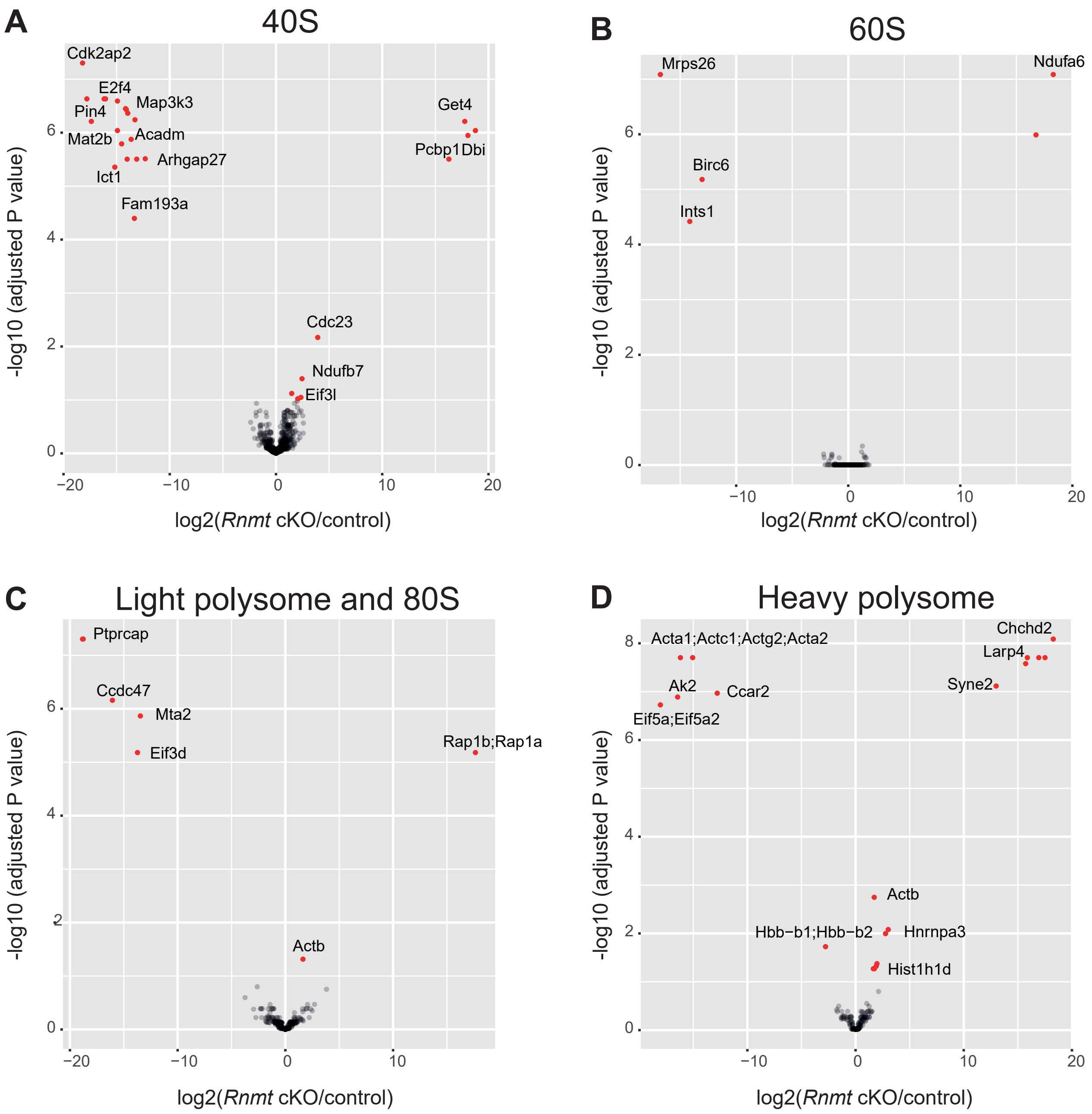

Supplemental figure S7. **Poly MEGA SEC proteomics analysis A-D)** Ribo Mega-SEC analysis of control (n=3) and *Rnmt* cKO (n=3) CD4 T cells, activated for 20 hours. **A)** 40S, **B)** 60S, **C)** light polysomes/80S and **D)** heavy polysomes were separated by Ribo Mega-SEC. Proteomics analysis of each fraction was normalised to the total signal and differential protein expression determined using linear modelling in Limma. Volcano plots from comparison of controls and *Rnmt* cKOs within each fraction are shown, each point represents a protein, significantly changed (adjusted p value <0.05) proteins in red, non-overlapping points are labelled with the protein identity.
