# Supplementary material for: Upregulation of RNA cap methyltransferase RNMT drives ribosome biogenesis during T cell activation
Source: Nucleic Acids Res. 2021 Jun 14;49(12):6722–38. doi: 10.1093/nar/gkab465 (PMC8266598; doi:10.1093/nar/gkab465)
Supplement: gkab465_Supplemental_Files [file gkab465_supplemental_files.zip › Supplementalmethodinformation.pdf]

## Supplemental Methods

### *In vitro* cap methyltransferase assay

N-7 cap guanosine methylation assay was performed according to (1). Extracts of naïve CD4 T cells and CD4 T cells activated for 20 hours with CD3 and CD28 were prepared. The N-7 cap guanosine methyltransferase activity of extracts from 40K naive cells and 17K activated cells were measured in each 10ul assay. Briefly, cell extracts were incubated with 200nM SAM and a $^{32}$P-m7G-capped substrate for 5, 10 and 20 mins. $^{32}$P-m7G-capped substrate was cleaved with P1 nuclease and caps resolved by thin layer chromatography. Caps were quantitated by phosphorimaging.

### *In vivo* cap methyltransferase assay

For the *in vivo* cap methylation assay (2), CD4 T cells were activated for 20 hours, the medium was replaced with methionine free medium (Methionine free RPMI (Gibco), 10% FCS, 50µM 2ME, pen/strep) supplemented with 1.5 MBq/ml L-[Methyl-3H]-Methionine (Perkin Elmer), cells were incubated at 37°C for 15 mins, then 5% T cell culture medium (containing methionine) was added to enhance survival and cells were incubated for a further 2 hours. mRNA was isolated as described above and was digested with 2 units of Nuclease P1 (Sigma) in nuclease P1 buffer (20mM NH4Oac pH 5.5) for 2 hours at 37°C, then with 10 units of Antarctic phosphatase (Thermo Fisher) for 3 hours at 37°C. The resulting nucleotide mixes were fractionated by anion exchange chromatography on a hiScreen CAPTO DEAE column (GE lifesciences) equilibrated with 50mM Tris pH 7.5. Samples were eluted with a linear gradient of NaCl from 0 to 1M with 50mM Tris pH 7.5. Prior to analysis of the samples, the standard compounds adenosine (Sigma), $^{m7}$GpppG (NEB), GpppG (NEB), and $^{m7}$GpppG$_m$ and GpppG$_m$ were injected and monitored by UV absorbance. 1ml fractions were collected and the fractions known to contain nucleotides were dried down to 100ul, mixed with 2ml Optiphase HiSafe 3 scintillation fluid (Perkin Elmer) and counted using a Tri-Carb 4910TR scintillation counter (Perkin Elmer).

### CAP-MAP mass spectrometry of cap structures

RNA was extracted from 20 hour activated CD4 T cells using Tri-reagent (Sigma), then mRNA purified using an mRNA Direct kit (ThermoFisher), binding and wash buffers were replaced with CAP-MAP binding buffer (1M $NH_4OAc$, 2mM EDTA) to reduce salt contamination in the final sample. mRNA was eluted from beads in water, then precipitated with 2.5M $NH_4OAc$, 1ul glycogen (Ambion) 1:1 isopropanol. Following washes in 75% Ethanol the mRNA was dried, dissolved in water, and quantitated using the qubit HS RNA assay (Thermo Fisher scientific). mRNA was digested with 9.5 units of Nuclease P1 (Sigma) in 20mM $NH_4OAC$ pH 5.3 for 3 hours at 37ºC. 250 fmols of ARCA was added, then LC-MS carried out as described.

CAP-MAP mass spec analysis was carried out as described previously with some alteration (3). Synthetic cap dinucleotide standards were either bought from New England Biolabs or synthesised. A list of the cap dinucleotides and their sources is available in Table 9. Syntheses of dinucleotide cap analogues GpppG$_m$, GpppA$_m$, Gppp$^{m6}$A$_m$, $^{m7}$GpppG$_m$, $^{m7}$GpppA$_m$, $^{m7}$Gppp$^{m6}$A, $^{m7}$Gppp$^{m6}$A$_m$, are described in the CAP MAP protocol. We also synthesised four new cap dinucleotides, $^{m7}$GpppU, $^{m7}$GpppC, $^{m7}$GpppC$_m$, and GpppC$_m$, using the same methodology as our previous publication (3). A chemically similar cap standard (ARCA ($^{m7}$G$_{O\text{-}3m}$pppG)) (NEB) was used as internal standard (Table 9).

The same LC-MS system, buffers and column were used as previously described (3). A modification of the gradient was required as the 4 new nucleotides used are more hydrophilic than those used previously. The column was equilibrated with 12% buffer B for 9 min at 0.04 ml/min and maintained at a constant temperature of 45ºC prior to sample injection. Aliquots of 14 µl of each sample were loaded onto the column and compounds were eluted with a linear gradient of 12–14% buffer B over 2 min, 14–40% buffer B over 2 min, 40–75% buffer B over 6 min, 75-85% buffer B over 4 min and finally 85-100% buffer B over 1 min The column was washed for 4 min in 100% buffer B before equilibration in 12% buffer B for 9 min.

Levels of the 15 cap nucleotides were measured using MRM mode with optimized collision energies and radio-frequencies previously determined by infusing pure compounds (Galloway et al., 2020). MS parameters for the 4 additional new cap nucleotides are listed in below.

| Compound | Precursor (m/z) | Product (m/z) | Collision Energy (V) | RF Lens (V) |
|---|---|---|---|---|
| GpppCm | 761.44 | 398 | 30 | 146 |

| | | | | |
|---|---|---|---|---|
| GpppCm | 761.44 | 424 | 28 | 146 |
| m7GpppC | 761.53 | 384.09 | 32 | 150 |
| m7GpppC | 761.53 | 595.95 | 22 | 150 |
| m7GpppU | 762.48 | 385 | 32 | 132 |
| m7GpppU | 762.48 | 597 | 22 | 132 |
| m7GpppCm | 775.63 | 397.96 | 33 | 130 |
| m7GpppCm | 775.63 | 610 | 23 | 130 |

A standard curve was prepared using 3–1000 fmol of the 15 cap dinucleotides with 250 fmol internal ARCA standard spiked into each sample. The standard curve was run prior to running the experimental samples using the same conditions, and was used to calculate the relative amount of the 15 cap nucleotides in each sample. One blank was run between each sample to eliminate carry-over.

**TMT proteomics**

Proteomics was done according to the method of (4). FACS sorted naive or 20h activated CD4 T cells were snap frozen on dry ice then lysed in proteomics lysis buffer (4% SDS, 10mM TCEP, 50mM TEAB) in protein LoBind Eppendorf tubes. Lysates were incubated at 99°C 750 rpm for 5 minutes in the thermomixer, cooled for 5 minutes, and sonicated with 15 cycles of 30s on/30s off. Nucleic acids were digested with Benzonase (Millipore) for 15 mins at 37°C. Proteins were quantitated using an EZQ Protein Quantitation Kit (Thermo Fisher Scientific). Iodoacetamide was added at a final concentration of 20mM and samples were incubated in the dark for 1 hour at room temperature. An equal mixture of hydrophilic and hydrophobic SP3 beads (GE healthcare) was added to bind proteins (5). Acentonitrile (ACN) and formic acid (FA) were added to give a final concentration of 50% ACN and 0.5% FA, and the sample was mixed for 8 minutes then beads washed once in 70% Ethanol and once in 100% ACN. Proteins were digested in 85µl digest buffer (0.1% SDS, 50mM TEAB, 1mM $CaCl_2$) with 1ug Trypsin (Pierce Trypsin Protease, MS Grade, Thermo Fisher scientific) overnight at 37°C, another 1µg of Trypsin was added then the sample incubated overnight at 37°C again. Peptides were quantified using a CBQCA assay (Thermo Fisher scientific), then labelled with a TMT six plex (naïve) or TMT 10 plex (activated) isobaric labelling set (Thermo Fisher scientific). TMT labelling reagents were dissolved in 41ul ACN and added to the peptide mixture in two 21ul batches 30 minutes apart, incubated for a further 30 mins, then the reaction stopped by adding 5µl of 5% hydroxylamine. Labelled peptides were bound to beads by adding 1900µl of ACN for 8 minutes, then magnetically separated, washed in ACN, dried, then eluted in 2% DMSO.

**High pH RP Fractionation**

Samples were re-dissolved in 200 µl ammonium formate ($NH_4HCO_2$) (10mM, pH 9.5) and peptides were fractionated using High pH RP Chromatography. A C18 Column from Waters (XBridge peptide BEH, 130Å, 3.5 µm 2.1 X 150 mm, Waters, Ireland) with a guard column (XBridge, C18, 3.5 µm, 2.1X10mm, Waters) were used on a Ultimate 3000 HPLC (Thermo-Scientific). Buffers A and B used for fractionation consist, respectively, of (A) 10 mM ammonium formate in milliQ water pH 9.5 and (B) 10 mM ammonium formate, pH 9.5 in 90% acetonitrile. Fractions were collected using a WPS-3000FC auto-sampler (Thermo-Scientific) at 1 minute intervals. Column and guard column were equilibrated with 2% Buffer B for twenty minutes at a constant flow rate of 0.2ml/min. 189 µl of TMT labelled peptides were injected onto the column, and the separation gradient was started 1 minute after sample was loaded onto the column. Peptides were eluted from the column with a gradient of 2% Buffer B to 20% Buffer B in 6 minutes, from 20% Buffer B to 45% Buffer B in 51 minutes and finally from 45% buffer B to 100% buffer B in 1 min. The column was washed for 15 minutes in 100% Buffer B and equilibrated at 2% Buffer B for 20 minutes as mentioned previously. The fraction collection started 1 minute after injection and stopped after 80 minutes (total 80 fractions, 200µl each). The total number of fractions concatenated was set to 20 and the content of the fractions was dried and suspended in 50µl of 1% formic acid prior to analysis with LC-MS.

**LC-MS Analysis**

Analysis of peptides was performed on a Q-exactive-HF (Thermo Scientific) mass spectrometer coupled with a Dionex Ultimate 3000 RS (Thermo Scientific). LC buffers were the following: buffer A (0.1% formic acid in Milli-Q water (v/v)) and buffer B (80% acetonitrile and 0.1% formic acid in Milli-Q water (v/v). Aliquots of 15 µL of each fraction were loaded at 10 µL/min onto a trap column (100 µm ×

2 cm, PepMap nanoViper C18 column, 5 µm, 100 Å, Thermo Scientific) equilibrated in 0.1% formic acid. The trap column was washed for 3 min at the same flow rate with 0.1% FA and then switched in-line with a Thermo Scientific, resolving C18 column (75 µm × 50 cm, PepMap RSLC C18 column, 2 µm, 100 Å). The peptides were eluted from the column at a constant flow rate of 300 nl/min with a linear gradient from 5% buffer B (for Fractions 1-10, 7% for Fractions 11-20) to 35% buffer B in 125 min, and then to 98% buffer within 2 min. The column was then washed with 98% buffer B for 20 min and re-equilibrated in 5% or 7% buffer B for 17 min. The column was kept all the time at a constant temperature of 50ºC.
Q-exactive HF was operated using an easy spray source operated in positive mode with spray voltage at 2.5 kV, the capillary temperature at 250ºC and the S-lens RF level at 60. A scan cycle comprised MS1 scan (activated CD4 T cell experiment: m/z range from 335-1600, naïve CD4 T cell experiment: m/z range from 335-1800), with default charge set to 2, maximum ion injection time 50 ms, a resolution of 120 000, automatic gain control (AGC) value of $3x10^6$ . The MS1 scan cycle was followed by 15 sequential dependant MS2 scans (activated CD4 T cell experiment: resolution 60000, naïve CD4 T cell experiment: resolution 15000) of the most intense ions fulfilling predefined selection criteria (activated CD4 T cell experiment: AGC $1x10^5$, maximum ion injection time 200 ms, isolation window 0.7 m/z, fixed first mass 100 m/z, spectrum data type: centroid, exclusion of unassigned, singly and >7 charged precursors, peptide match preferred, exclude isotopes on, dynamic exclusion time 45 s) (naïve CD4 T cell experiment: AGC 2x105, maximum ion injection time 80 ms, isolation window 0.4 m/z, fixed first mass 100 m/z, exclusion of unassigned, singly and >7 charged precursors, peptide match preferred, exclude isotopes on, dynamic exclusion time 45 s). The HCD collision energy was set to 32% of the normalized collision energy. Mass accuracy is checked before the start of samples analysis.

**Proteomics analysis**
Protein quantifications were calculated using Maxquant 1.5.8.3 using the *M. musculus* reviewed SwissProt data set (2017-03) for protein/peptide searches. From the protein groups Table the proteins that were contaminants, reversed, only identified by site, and only identified by one peptide were removed. Summed signals from histones were used to calculate the normalisation factors between samples, and the fold changes and statistical significance of changes were calculated using linear modelling in Limma (6). Plots were drawn in R using ggplot and hits with an FDR below 0.05 were submitted to Webgestalt for gene set overrepresentation analysis.

**RNA sequencing**
Naïve T cell transcriptome libraries were prepared by the Tayside Centre for Genomic Analysis using an Illumina Truseq kit with Ribozero Gold rRNA depletion. 20 hour activated T cell cytoplasmic transcriptome and ribosome footprint libraries were prepared using the Illumina Truseq Ribo Profile (mammalian) kit. 75bp paired-end sequencing was performed by the Tayside Centre for Genomic Analysis using an Illumina Nextseq. Sequence quality was checked using FastQC. For the ribosome footprinting analysis read two was selected for further processing since read one sequencing failed.

**Analysis of RNaseq**
Reads were aligned to mm10 with gencode vM12 basic annotation, and read counts for each gene calculated using STAR version 2.5.2b. Gene expression was compared between controls and *Rnmt* cKO in R using EdgeR, using a counts per million (CPM) threshold of 1 in at least three samples, scatterplots drawn using ggplot2, and hits with an FDR below 0.05 were submitted to Webgestalt for gene set overrepresentation analysis. Gene annotations were obtained through biomaRt.
Certain sRNAs have multiple copies, therefore, to assess sRNA expression, reads were realigned to mm10 using STAR 2.5.2b with filters on multimapping reads removed and gene counts recalculated with HTseq such that primary alignments of each multimapping read was counted. sRNA genes were grouped according to their RFAM family, and each RFAM family assigned the summed read counts from those genes. Overlaps between sRNAs and mRNAs/lncRNAs were identified using GenomicRanges in R. Differential expression analysis was repeated using EdgeR using a cpm threshold of 1 in at least three samples, mRNAs were included to allow total read count normalisation between libraries.
To assess splicing, a custom GFF annotation file, including intron and exon locations was generated using a perl script posted on seqanswers (http://seqanswers.com/forums/showthread.php?t=42420) by Alejandro Reyes, then reordered according to strand in R. Reads in each exon or intron of each gene were counted using HTSeq, and differential splicing analysis was carried out using DEXseq. Violin plots describing the overall trend in certain exon/intron positions were drawn using ggplot2.

**Analysis of ribosome footprinting**

Prior to alignment, adapter sequences (GATCGTCGGACTGTAGAACT) were removed using Cutadapt. Reads aligning to ribosomal RNA and tRNAs were filtered out using Bowtie, then the remaining reads were aligned to mm10 with Gencode vM12 basic annotation using STAR 2.5.2b. EdgeR was used for comparisons between control and KO total RNA and RPF RNAs, and Ribodiff was used to calculate and compare translation efficiencies. Scaled Venn diagrams were drawn using VennDiagram in R.

Splicing was assessed in the activated T cell total RNA library using the same method as the naïve T cell RNAseq.

**eCLIP**

eCLIP was carried out essentially as described in the seCLIP protocol with a few modifications (7). Magnet sorted lymph node CD4 T cells were suspended in PBS and exposed to 400mJ of 254nm UV then snap frozen on dry ice. Cells were thawed into lysis buffer (50 mM Tris-HCl pH 7.4, 100 mM NaCl, 1% NP-40 (Igepal CA630), 0.1% SDS, 0.5% sodium deoxycholate, 1:200 Protease Inhibitor Cocktail III (Sigma), 1:200 murine RNAse inhibitor (NEB)) and lysed on ice for 15 minutes. Lysates were incubated with 1µl/ml turbo DNAse (Thermo Fisher Scientific) and 1µl/ml of a 1:2000 (1:1000 in preliminary experiment Fig. 3G) dilution of RNAse1 (Thermo Fisher Scientific) in PBS for 5 mins at 37°C then returned to ice then centrifuged to remove insoluble material.

Lysates were precleared with protein G dynabeads (Thermo Fisher Scientific) at 4°C for 1hour. Lysates underwent immunoprecipitation with 50µl of protein G dynabeads preincubated with 4 µg of either LARP1, or isotype control antibody overnight at 4°C, 10% of the LARP1 samples were taken for the size matched input control. Beads were washed three times for 5 minutes with high salt wash buffer (50 mM Tris-HCl pH 7.4, 1 M NaCl, 1 mM EDTA, 1% NP-40, 0.1% SDS, 0.5% sodium deoxycholate) with 2M urea. For the ProteinTech and Santa Cruz Biotech antibodies (and their isotype controls) used in the preliminary experiment a medium salt buffer (50 mM Tris-HCl pH 7.4, 600mM NaCl, 1 mM EDTA, 1% NP-40, 0.1% SDS, 0.5% sodium deoxycholate) was used for these washes. Samples were then washed with lysis buffer, then TAP buffer (10 mM Tris pH 7.5, 5 mM MgCl2,100 mM KCl, 0.02% Triton X-100). RNA was dephosphorylated with FastAP Thermosensitive Alkaline Phosphatase (Thermo Fisher Scientific) at 1200 rpm, 37°C, then beads washed in wash buffer (20 mM Tris-HCl pH 7.4, 10 mM $MgCl_2$, 0.2% Tween-20, in water) twice. A 5% aliquot of the immunoprecipitate was labelled with gamma 32P-ATP (Perkin Elmer) using PNK (NEB) to check for RNA-protein complexes. For library construction, 2.5µl of the 3' RNA adapter InvRiL19 (40µM) (sequences in Table 9) was ligated to bead bound RNA fragments at room temperature for 75 minutes using the following mixture: 9µl $H_2O$, 3µl 10X ligase buffer (500 mM Tris-HCl pH 7.5, 100 mM MgCl2), 0.3 µL 0.1 M ATP, 0.8µl 100% DMSO, 9µl 50% PEG 8000, 0.4µl Murine RNase Inhibitor, 2.5 µL High concentration T4 RNA Ligase (NEB). Beads were then washed in wash buffer, high salt wash buffer, then wash buffer.

RNA-protein complexes were denatured in NuPage loading buffer (Thermo Fisher Scientific) + 50mM DTT, resolved 3-8% Tris Acetate gel (Thermo Fisher Scientific) in Tris Acetate SDS buffer (Thermo Fisher Scientific), and transferred to a nitrocellulose membrane in NuPage transfer buffer (Thermo Fisher Scientific). RNA protein complexes were identified from the 32P-ATP labelled membrane, and cut from the membrane containing libraries. RNA was released by incubating with Proteinase K for 20 minutes at 37°C, then with 420mg/ml urea in Proteinase K buffer for 20 minutes at 37°C and purified by phenol chloroform extraction.

The input library was dephosphorylated with FastAP Thermosensitive Alkaline Phosphatase (Thermo Fisher Scientific) at 1200 rpm, 37°C, then cleaned up with 20µl myOneSilane beads (Thermo Fisher Scientific) using RLT buffer (Qiagen), NaCl, and ethanol in the binding buffer, and 75% ethanol as a wash buffer then dissolved made up in 10µl water. For 3' adapter ligation 5µl of the input was mixed 0.5 µL InvRiL19 and 1.5µl DMSO then incubated for 75 minutes at room temperature with the following mixture: 1.5µl $H_2O$, 2µl 10X ligase buffer, 0.2 µL 0.1 M ATP, 0.3µl 100% DMSO, 8µl 50% PEG 8000, 0.2µl Murine RNase Inhibitor, 1.3 µL High concentration T4 RNA Ligase (NEB). Input samples were then cleaned up again with 20µl myOneSilane beads, with RLT buffer plus ethanol as the binding buffer and 75% ethanol as the wash buffer.

RNA was reverse transcribed using superscript III (Thermo Fisher Scientific), excess primers removed with EXOSAPIT (Thermo Fisher Scientific), and RNA removed by heating at 70°C for 12 minutes with 120mM NAOH, followed by neutralisation with HCl. cDNA was cleaned up with 10µl myOneSilane beads using RLT buffer (Qiagen) with ethanol as a binding buffer and eluted in 5 µL 5 mM Tris-HCl pH 7.5. 0.8µl of the 3' DNA linker InvRand3Tr3 and 1µl DMSO were added then ligated at room

temperature overnight with this mixture: 1.5µl High concentration T4 ligase (NEB), 2µl proprietary T4 ligase buffer, 0.2µl 0.1M ATP, 9µl of 50% PEG 8000, and 1.1µl $H_2O$.
Adapter linked cDNA was cleaned up with silane beads, and amplified by PCR with Phusion High-Fidelity DNA Polymerase (Thermo Fisher) using the HF buffer provided. PCR products were cleaned up with AMPure beads, and gel purified on an agarose gel, extracted with a Qiagen MinElute gel extraction kit. Libraries were quantified using the qbit HS DNA assay (Thermo Fisher) and quality checked using the tapestation HS DNA assay (Agilent).
Libraries were sequenced with a Nextseq High Output v2.5 kit (75 cycles, single end). The preliminary experiment was sequenced with MiSeq v3 (150-cycle, paired end- read one used)

**eCLIP analysis**
First the 10nt randomer barcode was extracted using umi_tools, then adapter sequences were trimmed from the reads using cutadapt. Reads were aligned to mm10 with Gencode vM12 basic annotation using STAR 2.5.2b, then demultiplexed using umi_tools to remove PCR duplicates. Secondary alignments and alignments with a MAPQ <20 were removed using samtools. Peaks were called using pureCLIP (options -iv 'chr1;chr2;chr3;chr4;chr5;' --mtc 10000 --ctr --dm 30) using the size matched input control for the input. The binding regions were subjected to further analysis- first the reads aligning to each peak were calculated using countOverlaps from the GenomicRanges R package. The ratio of reads in the LARP1 eCLIP sample to the input and to the IgG control were calculated, 0.25 was added to the input and IgG controls to avoid infinite ratios. Peaks with a LARP1 eCLIP: input read ratio greater than or equal to 5 and a LARP1 eCLIP: IgG read ratio greater than or equal to 10 were selected for further analysis, a few peaks from the Y chromosome were also removed (mice were female). Control mouse and *Rnmt* cKO peaks were combined and overlapping peaks merged using GenomicRanges::reduce.
Peaks were annotated using the Encode vM25 basic annotation using the RCAS package in R. Annotations were simplified by removing transcript, exon, and gene coordinates for protein coding genes, leaving 5'UTR, CDS, intron and 3'UTR. For non-coding transcripts "transcribed_processed_pseudogene", "polymorphic_pseudogene" and "processed_pseudogene" were grouped as "pseudogene"; and "processed_transcript", "antisense", "bidirectional_promoter_lncRNA" and "lincRNA" were grouped as "lncRNA". Where peaks did not overlap with an annotated transcript, they were assigned to overlapping transcripts within 100bp downstream of the peak to allow for annotation errors, those that still did not overlap with a feature were labelled intergenic. A list of snoRNAs hosts overlapping with LARP1 binding sites was generated and a list of snoRNAs overlapping with these hosts generated using GenomicRanges::subsetByOverlaps. mRNAs with binding in the 5'UTR were split into pathways for further analysis, this was done manually, pathways are indicated for the relevant genes in Table 2. 15bp regions centred on the start of each 5'UTR associated peak were generated and the sequences extracted, the proportion of bases at each position was plotted using ggseqlogo.
A GTF file was written with the transcript coordinates for protein coding genes with LARP1 binding sites, where multiple transcripts from the same gene overlapped with a LARP1 binding site, the first listed was selected. A bed file was written with the gene coordinates of LARP1 target pseudogenes and lncRNAs. These annotation files were used to draw profiles of LARP1 binding in deeptools. Coverage for LARP1 eCLIP control and *Rnmt* cKO libraries was calculated using bamCoverage -bs 10 --Offset 1 1 --normalizeUsing CPM –exactScaling. Matrices calculated using computeMatrix, the metagene option was used for protein coding genes- this calculates coverage over exon regions for each transcript, for non-coding genes coverage over the genes was calculated. plotProfile was used to generate the plots.
LARP1 eCLIP data for individual genes/binding sites was plotted using GVIZ in R. For comparison, C57Bl/6J CD4 T cell CAGE data, in BAM format, was downloaded from the FANTOM5 project, CAGE data indicates transcript start sites, which are where cap dependent LARP1 binding is expected. CLIP reads and CAGE data were visualised using GVIZ in R- the position of the first nucleotide of each read was plotted for both these data types.

**Pseudouridine sequencing**
300ng of total RNA from each replicate was made up in BEU buffer (50mM bicine pH 8.3, 4mM EDTA, 7M urea) for the mock treated sample or BEU buffer plus 0.17M CMC (N-Cyclohexyl-N′-(2-morpholinoethyl)carbodiimide methyl-p-toluenesulfonate) (Sigma). Samples were heated to 37°C for 20 mins, then RNA precipitated by adding 100ul buffer A (0.3M NaOAc, 0.1mM EDTA), 1 ul glycogen and 700ul ethanol and washed 1X in 75% EtOH. Samples were resuspended in buffer A, then precipitated as before with ethanol to remove any remaining CMC. RNA was made up in 50mM

sodium bicarbonate pH10.4 and heated to 37°C for 3 hours (this removes unwanted CMC adducts). RNA was precipitated with 100ul buffer A, 1ul glycogen, 700ul Ethanol, and washed in 75% ethanol. RNA was made up in 50mM sodium bicarbonate pH9.2 and heated to 95C for 8 mins to fragment, then onto ice. RNA was precipitated with 200ul buffer A, 1 ul glycogen, 500ul ethanol, and washed in 75% ethanol.
The libraries were prepared from this RNA as per the input libraries for eCLIP (see above), with the modification that 0.5ul High concentration T4 ligase (NEB) was used during ligation steps.
Libraries were sequenced with a Nextseq High Output v2.5 kit (75 cycles, single end).

**Pseudouridine sequencing analysis**
First the 10nt randomer barcode was extracted using umi_tools, then adapter sequences were trimmed from the reads using cutadapt. Reads were aligned to a customised Fa file containing rRNA sequences available from GSE128947:Supplementary_Data_1.fa using bowtie2, then demultiplexed using umi_tools to remove PCR duplicates (8). Secondary alignments and alignments with a MAPQ <20 were removed using samtools. The PSU scores were calculated using the GenomicRanges:countOverlaps tool in R. The % termination score is calculated as the reads beginning at a nucleotide (countOverlaps option type="start") divided by all the reads covering that nucleotide (countOverlaps option type="any") times 100. The CMC treated % termination score is divided by the mock treated % termination score to give the PSU score. The scores are then applied to the nucleotides 1 position upstream (since the final nucleotide reverse transcribed is the one before the CMC-adduct). To call a pseudouridine, a threshold of 3% for the percent termination, a 3 for the PSU score was used. Control and *Rnmt* cKO PSU scores were compared by linear modelling in limma using the eBayes test, but no significant differences were found. Plots were generates using ggplot2 in R.

**RNAPII ChIPseq analysis**
SRA tools were used to download the fastq files containing the following CHIPseq data: RNAPII (RNAPII: GSM1903984, input: GSM1903982, (9)). Reads were aligned to mm10 using STAR 2.5.2b. For comparison of RNAPII CHIP data and naïve T cell RNAseq, the raw counts of for each gene RNAPII or input were calculated in seqmonk with a +/- 100 bp window surrounding the gene. Genes with fewer than 10 reads in the input were removed and the log2 RNAPII/input ratio was calculated in R, then compared to naïve T cell data, the plot was drawn in ggplot2.

**RNMT-RAM KD HeLa data**
For analysis of RPG expression in RNMT-RAM KD HeLa cells, data from (10) supplemental data 1 was redrawn in R using ggplot2 with RPGs highlighted.
sRNA expression was analysed the similarly to the CD4 T cell data: reads (GSE87767) were realigned to hg38 using STAR 2.5.2b with filters on multimapping reads removed and gene counts recalculated with HTseq such that primary alignments of each multimapping read was counted. sRNA genes were grouped according to their RFAM family, and each RFAM family assigned the summed read counts from those genes. Overlaps between sRNAs and mRNAs/lncRNAs were identified using GenomicRanges in R. Differential expression analysis was repeated using EdgeR including mRNAs to allow total read count normalisation between libraries.

**Gene lists**
In various analyses of RNAseq or proteomics data, lists of genes have been highlighted. These included:

- The list of TOP RNAs is from (11) supplemental material 4.
- The Dang Myc upregulated list (12) was downloaded from the GSEA molecular signatures database.
- The Eick Myc induced ribosome processing genes are from (13)

**Ribo Mega-SEC**
Ribo Mega-SEC for the separation of polysomes and ribosomal subunits using size exclusion chromatography was performed as previously reported (14) with a slight modification. Briefly, $3.9 \times 10^6$ cells were lysed by vortexing for 10 sec in 75 µl of polysome extraction buffer (20 mM Hepes-NaOH (pH 7.4), 130 mM NaCl, 10 mM $MgCl_2$, 5% glycerol, 1% CHAPS, 0.2 mg/ml heparin, 2.5 mM DTT, 20 U SUPERase In RNase inhibitor, cOmplete EDTA-free Protease inhibitor), incubated for 15 min on ice, and centrifuged at 17,000 g for 10 min. Supernatants were filtered through 0.45 µm Ultrafree-MC HV centrifugal filter units (Millipore).

Using a Dionex Ultimate 3,000 Bio-RS uHPLC system (Thermo Fisher Scientific), a SEC column (Agilent Bio SEC-5, 2,000 Å pore size, 7.8 × 300 mm with 5 μm particles) was equilibrated with three column volumes of filtered SEC buffer (20 mM Hepes-NaOH (pH 7.4), 60 mM NaCl, 10 mM $MgCl_2$, 0.3% CHAPS, 0.2 mg/ml heparin, 2.5 mM DTT, 5% glycerol) (all column conditioning and separation at 5°C). 60 μl of the filtered cell lysates was injected onto the pre-equilibrated SEC column. The flow rate was 0.8 ml/min and the chromatogram was monitored by measuring UV absorbance at 215, 260 and 280 nm with a 1 Hz data collection rate by the Diode Array Detector. 10 × 600 μl fractions were collected from 7.75 min to 15.25 min using a low-protein binding 96-deep-well plate 1 mL (Eppendorf) at 4°C.

**Proteomics of Ribo Mega-SEC fractions**

600 ml of heavy polysomes, light polysomes/ 80S, 60S, or 40S fraction was treated with Benzonase for 15 mins at 37°C. Proteins were then reduced using TCEP (25 mM final concentration) for 15 min at 65°C and alkylated using N-Ethylmaleimide (25 mM final concentration) in the dark for 1 hour at room temperature. Protein purification, digestion and peptide purification were performed by SP3 as described above. Purified peptides were resuspended in 50 μl of 1% formic acid and aliquots of 15 μl were analysed by Q-exactive plus (Thermo Scientific) mass spectrometer with the same LC setting described as above. Q-exactive plus was operated using an easy spray source operated in positive mode with spray voltage at 2.5 kV, the capillary temperature at 250ºC and the S-lens RF level at 60. A scan cycle comprised MS1 scan (m/z range from 350-1,600), with default charge set to 2, maximum ion injection time 20 ms, a resolution of 70,000, automatic gain control (AGC) value of $1x10^6$ . The MS1 scan cycle was followed by 15 sequential dependant MS2 scans (resolution 17,500) of the most intense ions fulfilling predefined selection criteria (AGC $1x10^5$, maximum ion injection time 60 ms, isolation window 1.4 m/z, fixed first mass 140 m/z, spectrum data type: profile, exclusion of unassigned, singly and >7 charged precursors, peptide match preferred, exclude isotopes on, dynamic exclusion time 40 s). The HCD collision energy was set to 27% of the normalized collision energy. Mass accuracy is checked before the start of samples analysis.

**Ribo Mega-SEC data analysis**

Raw data was analysed in Maxquant using the mouse Swissprot database for peptide identification. iBAQ scores for each protein were used for further analysis in R. The datasets were analysed with limma using the sum of iBAQ scores for normalisation and fold changes and statistical significance were calculated for comparisons between control and *Rnmt* cKO samples within each fraction. Graphs were drawn using ggplot2.


1. Varshney, D., Petit, A.P., Bueren-Calabuig, J.A., Jansen, C., Fletcher, D.A., Peggie, M., Weidlich, S., Scullion, P., Pisliakov, A.V. and Cowling, V.H. (2016) Molecular basis of RNA guanine-7 methyltransferase (RNMT) activation by RAM. *Nucleic Acids Res*, **44**, 10423-10436.
2. Belanger, F., Stepinski, J., Darzynkiewicz, E. and Pelletier, J. (2010) Characterization of hMTr1, a human Cap1 2'-O-ribose methyltransferase. *The Journal of biological chemistry*, **285**, 33037-33044.
3. Galloway, A., Atrih, A., Grzela, R., Darzynkiewicz, E., Ferguson, M.A.J. and Cowling, V.H. (2020) CAP-MAP: cap analysis protocol with minimal analyte processing, a rapid and sensitive approach to analysing mRNA cap structures. *Open Biol*, **10**, 190306.
4. Sinclair, L.V., Howden, A.J., Brenes, A., Spinelli, L., Hukelmann, J.L., Macintyre, A.N., Liu, X., Thomson, S., Taylor, P.M., Rathmell, J.C. *et al.* (2019) Antigen receptor control of methionine metabolism in T cells. *Elife*, **8**.
5. Hughes, C.S., Foehr, S., Garfield, D.A., Furlong, E.E., Steinmetz, L.M. and Krijgsveld, J. (2014) Ultrasensitive proteome analysis using paramagnetic bead technology. *Mol Syst Biol*, **10**, 757.
6. D'Angelo, G., Chaerkady, R., Yu, W., Hizal, D.B., Hess, S., Zhao, W., Lekstrom, K., Guo, X., White, W.I., Roskos, L. *et al.* (2017) Statistical Models for the Analysis of Isobaric Tags Multiplexed Quantitative Proteomics. *J Proteome Res*, **16**, 3124-3136.
7. Van Nostrand, E.L., Nguyen, T.B., Gelboin-Burkhart, C., Wang, R., Blue, S.M., Pratt, G.A., Louie, A.L. and Yeo, G.W. (2017) Robust, Cost-Effective Profiling of RNA Binding Protein Targets with Single-end Enhanced Crosslinking and Immunoprecipitation (seCLIP). *Methods Mol Biol*, **1648**, 177-200.

8. Hebras, J., Krogh, N., Marty, V., Nielsen, H. and Cavaille, J. (2020) Developmental changes of rRNA ribose methylations in the mouse. *RNA Biol*, **17**, 150-164.
9. Onodera, A., Tumes, D.J., Watanabe, Y., Hirahara, K., Kaneda, A., Sugiyama, F., Suzuki, Y. and Nakayama, T. (2015) Spatial Interplay between Polycomb and Trithorax Complexes Controls Transcriptional Activity in T Lymphocytes. *Mol Cell Biol*, **35**, 3841-3853.
10. Varshney, D., Lombardi, O., Schweikert, G., Dunn, S., Suska, O. and Cowling, V.H. (2018) mRNA Cap Methyltransferase, RNMT-RAM, Promotes RNA Pol II-Dependent Transcription. *Cell Rep*, **23**, 1530-1542.
11. Thoreen, C.C., Chantranupong, L., Keys, H.R., Wang, T., Gray, N.S. and Sabatini, D.M. (2012) A unifying model for mTORC1-mediated regulation of mRNA translation. *Nature*, **485**, 109-113.
12. Zeller, K.I., Jegga, A.G., Aronow, B.J., O'Donnell, K.A. and Dang, C.V. (2003) An integrated database of genes responsive to the Myc oncogenic transcription factor: identification of direct genomic targets. *Genome Biol*, **4**, R69.
13. Schlosser, I., Holzel, M., Murnseer, M., Burtscher, H., Weidle, U.H. and Eick, D. (2003) A role for c-Myc in the regulation of ribosomal RNA processing. *Nucleic Acids Res*, **31**, 6148-6156.
14. Yoshikawa, H., Larance, M., Harney, D.J., Sundaramoorthy, R., Ly, T., Owen-Hughes, T. and Lamond, A.I. (2018) Efficient analysis of mammalian polysomes in cells and tissues using Ribo Mega-SEC. *Elife*, **7**.
